# Licorice isoliquiritigenin-encapsulated mesoporous silica nanoparticles for osteoclast inhibition and bone loss prevention

**DOI:** 10.7150/thno.33376

**Published:** 2019-07-09

**Authors:** Xiaoyue Sun, Jie Zhang, Zijun Wang, Bingqian Liu, Shenting Zhu, Lingxin Zhu, Bin Peng

**Affiliations:** The State Key Laboratory Breeding Base of Basic Science of Stomatology (Hubei-MOST) & Key Laboratory of Oral Biomedicine Ministry of Education, School & Hospital of Stomatology, Wuhan University, Wuhan 430079, China.

**Keywords:** mesoporous silica nanoparticles, isoliquiritigenin, drug delivery, osteoclast, lipopolysaccharide

## Abstract

Mesoporous silica nanoparticles (MSNs) are extensively used in bone tissue regeneration and local drug delivery. However, the effects of MSNs alone on osteoclast formation and function, as well as the utilization of MSNs to deliver natural molecules against bone resorption, remain unexplored. Here, we report the development of licorice-derived bioactive flavonoid isoliquiritigenin (ISL)-encapsulated MSNs (MSNs-ISL) as a potent bone-bioresponsive nanoencapsulation system for prevention of osteoclast-mediated bone loss* in vitro* and* in vivo*.

**Methods**: We synthesized MSNs-ISL and then investigated the drug loading and release characteristics of the resulting nanoparticles. *In vitro* experiments on osteoclast differentiation and bone resorption were performed using mouse primary bone marrow-derived macrophages (BMMs). *In vivo* animal experiments were conducted using a lipopolysaccharide (LPS)-mediated calvarial bone erosion model.

**Results**: The resulting MSNs-ISL were spherical and highly monodispersed; they possessed a large specific surface area and superior biocompatibility, and allowed acid-sensitive sustained drug release. Compared with free ISL and MSNs alone, MSNs-ISL significantly and additively inhibited receptor activator of nuclear factor-κB ligand (RANKL)-induced osteoclast generation, decreased the size and quantity of sealing zones, and reduced the osteolytic capacity of osteoclasts *in vitro*. MSNs-ISL treatment also downregulated RANKL-stimulated mRNA expression of osteoclast-associated genes and transcription factors. Mechanistically, MSNs-ISL remarkably attenuated the RANKL-initiated expression of tumor necrosis factor receptor-associated factor 6 (TRAF6), phosphorylation of mitogen-activated protein kinases (MAPKs), and phosphorylation and degradation of inhibitor of κBα (IκBα), together with the nuclear translocation of nuclear factor-κB (NF-κB) p65 and the activator protein (AP)-1 component c-Fos. Moreover, MSNs-ISL almost completely restrained the expression of nuclear factor of activated T cells (NFATc1). Consistent with the *in vitro* results, MSNs-ISL could block osteoclast activity; relieve inflammation-related calvarial bone destruction *in vivo*; and suppress c-Fos, NFATc1, and cathepsin K expression levels.

**Conclusion**: Licorice ISL-encapsulated MSNs exhibit notable anti-osteoclastogenetic effects and protect against inflammatory bone destruction. Our findings reveal the feasibility of applying MSNs-ISL as an effective natural product-based bone-bioresponsive nanoencapsulation system to prevent osteoclast-mediated bone loss.

## Introduction

Osteoclasts are multinucleated cells that are specifically present in bone; these cells are responsible for osteolysis 1. Inflammation-induced processes in bone caused by pathogen exposure can induce excessive osteoclasts formation; including osteomyelitis, septic arthritis, and periodontitis 2. Therefore, osteoclasts serve as effective targets for drug development against lytic bone diseases.

Increasing attention has been given to develop biologically active flavonoids separated from traditional botanical drugs to control bone metabolism 3, 4. Isoliquiritigenin (ISL), also known as 2′,4′,4-trithydroxychalcone, is a natural flavonoid extracted from licorice. This flavonoid has attracted wide research interest on account of its pharmacological safety and biological properties, including antioxidative, antiviral, antidiabetic, anticancer, estrogenic, and immunomodulatory activities 5-7. The modulatory effects of ISL on bone homeostasis have been studied, and several studies, including our previous studies, have revealed that this substance can significantly attenuate osteoclastogenesis and has potential preventive effects on lytic bone diseases 8-10. However, despite its apparent benefits, the disadvantages of ISL, including poor solubility, short elimination half-life, and low bioavailability, greatly limit its applications in biomedical and clinical fields 11.

Nanotechnology has been increasingly applied to the biomedical field, particularly for the effective delivery of bioactive flavonoids 12-14. Mesoporous silica nanoparticles (MSNs) are extensively used as a superior drug delivery vehicle due to their various advantages, including stable physicochemical properties, high biocompatibility, efficient drug-loading rate resulting from their large surface area and high pore volume, and easy functionalization 15-17. MSNs have been reported to successfully improve the solubility and bioavailability of flavonoids with poor solubility, such as quercetin, baicalein, and curcumin, thereby enhancing their targeted delivery and therapeutic efficacy against cancer and/or inflammation 18-20. Two distinct forms of MSN-based composite materials, namely, magnetic mesoporous silica nanochains and MSNs/hydroxyapatite composite-coated implants, have recently been reported to inhibit osteoclast activity 21, 22. However, the effect of the universal and extensive forms of MSNs alone on the fusion and function of osteoclasts, the underlying signaling mechanisms, and the utilization of MSNs to deliver natural molecules against inflammatory osteolysis remain unexplored.

Given the intrinsic limitations of natural ISL for biomedical applications, we encapsulated ISL into MSNs, and evaluated the physical properties and the loading and release profiles of the resulting nanoparticles. We then comprehensively studied the effects and molecular mechanism of MSNs alone and ISL-encapsulated MSNs (MSNs-ISL) on nuclear factor-κB ligand (RANKL)-induced osteoclastogenesis *in vitro* and inflammation-related bone resorption* in vivo*.

## Methods

### Synthesis of MSNs-ISL

MSNs were prepared in the nonpolar core of micelles formed by water, surfactant and an organic solvent. We ameliorated the method of Kim for synthesizing MSNs 23. Cetyltrimethyl ammonium bromide (CTAB; 1 g) was added to 150 mL of distilled water and 40 mL of 2-ethoxyethanol. After homogenization, 2 mL of aqueous ammonia (28 wt %) was added to the mixed solution, and the mixture was heated to 80 °C. Tetraethyl orthosilicate (TEOS; 2 mL) was added dropwise to this mixture under 4 h of vigorous stirring to generate a white product, which was collected and calcined for 5 h at 550 ℃. The final product was dried under vacuum at -80 °C after rinsing with ethanol and distilled water to obtain MSNs.

MSNs-ISL were prepared according to the previous literature on flavonoid encapsulation with some modifications 20. ISL (20 mg) was added to dimethyl sulfoxide (DMSO; 1 mL). In another container, MSNs (20 mg) were dispersed in distilled water (1 mL). The two solutions were mixed and stirred in the dark at 37 ℃ for 24 h. After centrifugation, the products were collected and divided into two parts: one part was dried under vacuum at -80 °C and the other part was washed with a 1:1 mixture of DMSO:distilled water. All washing liquids were collected. Thereafter, a 0.22 μm syringe filter (Millipore, MA, USA) was used to filter the product, and the final product was dried under vacuum at -80 °C to obtain MSNs-ISL.

### Characterization of MSNs-ISL

The morphology of MSNs was recorded using a JEM-2100 transmission electron microscope (TEM) at an accelerating voltage of 200 kV (JEOL, Japan). Zeta potentials and particle size were analyzed using a Zetasizer Nano ZSP instrument (Malvern, UK). N2 adsorption-desorption measurement was performed according to the Brunauer-Emmet-Teller (BET) and Barrett-Joyner-Halenda (BJH) methods using a BELSORP-max instrument (MicrotracBEL Corp., Japan). Fourier transform infrared spectroscopy (FTIR) was conducted using a NICOLET 5700 FTIR spectrometer (Thermo Nicolet Corporation, USA). Thermogravimetric analysis (TGA) was performed using a STA7300 Thermo gravimetric analyzer (Hitachi Ltd. Japan). X-ray diffraction (XRD) was conducted on an X'Pert Pro X-ray diffractometer (PANalytical, The Netherlands). Ultraviolet-visible spectroscopy (UV-Vis) was conducted using a UV-2550 UV-Vis Spectrophotometer (Shimadzu, Japan).

### Loading and release efficacies of ISL

Two assessments of loading efficiency were undertaken. In the “wet” approach, the filtrate was collected and the liquid concentration was measured at 360 nm via UV-Vis spectroscopy. This assessment is required to calculate the encapsulation efficiency (EE). In the “dry” approach, the mass reductions of MSNs and MSNs-ISL after cyclic heating via TGA were compared to calculate the loading efficiency (LE).

LE (%) = (W_drug in NP_/W_NP+ drug in NP_) × 100

EE (%) = (W_drug in NP_/W_total drug_) × 100

Phosphate buffered saline (PBS; pH = 7.4 or 5.5) was used as a simulated environment to assess the release ability of ISL *in vitro*; 0.1% Tween 80 was added to dissolve ISL in PBS 24. The drug-loaded MSNs (1 mg) were placed in a 10, 000 Da dialysis bag (Beijing Solarbio Science & Technology Co., Ltd., China), immersed in the release medium (20 mL), and then slow stirred at room temperature. At specific intervals, the release medium (5 mL) was removed to calculate the ISL concentration via UV-Vis spectroscopy. The sample removed was substituted with an equivalent volume of fresh medium to maintain sink conditions. Cumulative release rates (%w/w) were calculated considering the actual encapsulation of ISL into MSNs. XRD was conducted on ISL crystals, MSNs, unwashed MSNs-ISL, and washed MSNs-ISL.

### Synthesis of rhodamine B (RhB)-labeled MSNs

RhB-labeled MSNs were synthesized as previously described 25. RhB was dissolved in distilled water to prepare an aqueous solution of RhB (1 mg/mL). MSNs (30 mg) were added to 8mL of the aqueous RhB solution, and the mixture was stirred in the dark at 37 ℃ for 48 h. After washing with distilled water, the product was collected by centrifugation. The final product was dried under vacuum at -80 °C to obtain MSNs-RhB.

### Animals

C57/BL6 mice (male; body weight: 18-20 g; age: 6-8 weeks) were purchased from the Experimental Animal Center of Hubei Province (Wuhan, China), and were placed in pressurized and ventilated cages. All animal studies were conducted under the guidelines of the Animal Welfare Act and the Guide for the Care and Use of Laboratory Animals by the Ethics Committee of the School and Hospital of Stomatology of Wuhan University, Wuhan, China.

### Cell culture

The femurs and tibias of C57/BL6 mice were washed with α-modified Eagle's medium (α-MEM; Gibco BRL, USA) to acquire bone marrow cells. After red blood cell lysis, the bone marrow cells were cultured in α-MEM supplemented with macrophage colony-stimulating factor (10 ng/mL, M-CSF; PeproTech, London, UK). After overnight culture, non-adherent cells were collected and transferred to medium containing M-CSF (30 ng/mL) for 5 days. Adherent cells were considered bone marrow-derived macrophages (BMMs).

### Cytotoxicity assay

BMMs were seeded into 96-well plates at a density of 1 × 10^4^ cells/well and allowed to adhere overnight. Different MSNs or MSNs-ISL concentrations (i.e., 16 and 64 μg/mL) with 30 ng/mL M-CSF were added, and the cells were cultured for the indicated time points (24, 48, or 72 h). Cell viability was evaluated using Cell Counting Kit-8 (CCK-8; Dojindo, Kumamoto, Japan) according to the manufacturer's protocol. Cells were incubated with 100 μL of fresh α-MEM containing 10 μL of CCK-8 reagent, and the absorbance of all wells at 450 nm was assessed using an ELISA microplate reader (Bio-Tek Instruments Inc., Winooski, VT, USA).

### TRAP staining

BMMs were seeded onto glass coverslips in 24-well plates at a density of 4×10^4^ cells/well. Then, either ISL (1 or 4 μg/mL), MSNs (16 or 64 μg/mL), or MSNs-ISL (16 or 64 μg/mL) was added to the complete medium plus 30 ng/mL M-CSF and 50 ng/mL RANKL, and only M-CSF and RANKL were added during medium change. After 7 days of culture, the cells were fixed with 4% paraformaldehyde and stained using a TRAP kit (Sigma-Aldrich, St Louis, MO) following the manufacturer's protocol. TRAP-positive cells containing three or more nuclei were counted as osteoclasts and quantified.

### Observation of F-actin rings

After culture treatment, BMMs were fixed with 4% paraformaldehyde and incubated in 0.1% Triton-PBS for 15 min to permeabilize the membrane. Cells were then blocked with normal goat serum (ZSGB-BIO, Beijing, China). F-actin rings were stained with FITC-labeled phalloidin (Sigma, St. Louis) for 45 min. After washing, the glass coverslips were infiltrated with mounting medium containing DAPI (nuclear dye; ZSGB-BIO, Beijing, China), and F-actin rings were observed and photographed using a fluorescence microscope (Leica, DMLS, Vienna, Austria).

### Intracellular uptake of MSNs-RhB

BMMs were seeded on glass coverslips in 24-well plates at a suitable density and incubated in complete culture medium with 30 ng/mL M-CSF and 50 ng/mL RANKL. Either BMMs or osteoclasts were treated with 100 μg/mL MSNs-RhB for 12 h. The cells were then fixed with 4% paraformaldehyde and the F-actin rings were stained. The glass coverslips were infiltrated with mounting medium containing DAPI (nuclear dye; ZSGB-BIO, Beijing, China). Intracellular uptake of MSNs-RhB was observed using a confocal laser scanning microscopy (CLSM; OLYMPUS, Japan).

### Observation of bone resorption pits

BMMs were seeded into a Corning osteo assay surface multiple well plate (Corning, NY, USA) at a density of 4×10^4^ cells/well. Either ISL (1 or 4 μg/mL), MSNs (16 or 64 μg/mL), or MSNs-ISL (16 or 64 μg/mL) was added, along with complete medium plus 30 ng/mL M-CSF and 50 ng/mL RANKL to the well. Seven days after culture, the cells were completely removed using 10% NaClO for 5 min at room temperature, and the plates were allowed to air dry at room temperature for 5 h. Bone resorption capacity was observed under an inverted microscope, and the percentage of surface area absorption on the plate was quantitatively analyzed.

### RNA extraction and qRT-PCR analysis

BMMs were seeded into six-well plates at the appropriate density. After 24, 48, or 72 h of stimulation with 30 ng/mL M-CSF and 50 ng/mL RANKL plus either MSNs (64 μg/mL) or MSNs-ISL (64 μg/mL), total cellular RNA was extracted using TRIzol reagent (Invitrogen, Carlsbad, CA). Exactly 1 µg of total RNA was reverse transcribed into cDNA using Oligo dT and ReverTra Ace following the manufacturer's instructions (Toyobo, Osaka, Japan). qRT-PCR analysis was performed on an ABI PRISM 7500 sequence detection system (Applied Biosystems, Foster City, CA) using a SYBR Green real-time PCR kit (Invitrogen, Carlsbad, CA). GAPDH was used as the internal control to determine RNA expression and the standard for gene expression quantification. The mRNA expression of each group was calculated as the ratio of the target mRNA to GAPDH. The specific primer sequences used for PCR are listed in Table S2 of the Supplementary Materials.

### Protein extraction and western blot analysis

BMMs were seeded into six-well plates at the appropriate density and stimulated with 30 ng/mL M-CSF and 50 ng/mL RANKL plus either MSNs (16 or 64 μg/mL) or MSNs-ISL (16 or 64 μg/mL) for 30 min or 24 h. The cells were washed twice with ice-cold PBS and then lysed using RIPA lysate containing protease and phosphatase inhibitor. Protein content was determined using a BCA protein quantification kit (Beyotime Inc, Shanghai, China) following the manufacturer's protocol. Total proteins were transferred onto a polyvinylidene difluoride membrane, blocked in non-fat milk for 1 h at room temperature, and incubated overnight with the specific primary antibodies at 4 °C. GAPDH served as the protein internal standard and the standard for quantifying protein expression. The specific primary antibodies used were p38, p-p38, extracellular signal-regulated protein kinase (ERK), p-ERK, nuclear factor-κB (NF-κB) p65, p NF-κB p65, inhibitor of κBα (IκBα), p-IκBα, GAPDH (Cell Signaling Technology, Danvers, MA), c-Jun N-terminal kinase (JNK), p-JNK, tumor necrosis factor receptor-associated factor 6 (TRAF6), and nuclear factor of activated T cells (NFATc1; Santa Cruz Biotechnology, Santa Cruz, CA). The gray values of the band were quantified.

### Immunofluorescence analysis

BMMs were seeded onto glass coverslips in 24-well plates at the appropriate density and cultured with either MSNs (64 μg/mL) or MSNs-ISL (64 μg/mL) in complete medium plus 30 ng/mL M-CSF and 50 ng/mL RANKL. After appropriate treatment, the cells were blocked for 1 h with normal goat serum (ZSGB-BIO, Beijing, China) at room temperature and incubated overnight at 4 °C with primary c-Fos and NF-κB p65 antibodies (Santa Cruz Biotechnology, CA). The cells were washed and incubated separately with Dylight 594- and 488-conjugated goat anti-mouse immunoglobulin G (Abbkine, California, USA) at 37 °C. The glass coverslips were then coated with mounting medium containing DAPI (nuclear dye, ZSGB-BIO, Beijing, China). Cells were imaged using a fluorescence microscope (Leica, DMLS, Vienna, Austria) and the intensity of immunofluorescence was quantified.

### Tissue distribution of MSNs-RhB *in vivo*

Healthy (age, 6-8 weeks) C57/BL6 mice were randomly divided into two groups (*n* = 6): (1) PBS, (2) MSNs-RhB. Mice were subcutaneously injected with MSNs-RhB (50 mg/kg body weight) at the midline of the calvarial sagittal suture. After three hours, the mice were sacrificed to collect calvarial bone, hearts, lungs, spleens, livers, and kidneys. Fluorescence signals were analyzed via biophotonic imaging (CRi maestro, USA). The calvaria were fixed in 4% paraformaldehyde for 2 days and then decalcified at 4 °C in 10% ethylenediaminetetraacetic acid (EDTA, pH 7.4) for 20 days. The tissues were embedded in optimal cutting temperature (OCT) compound (SAKURA Tissue-Tek, USA) and then sliced into 5 μm cryosections. After washing thrice with PBS, the cryosections were blocked with normal goat serum (ZSGB-BIO, Beijing, China) for 1 h at 37 °C and then incubated with FITC-labeled phalloidin (Sigma, St. Louis) for 40 min. The cryosections were mounted in mounting medium containing DAPI (nuclear dye, ZSGB-BIO, Beijing, China) and observed under a fluorescence microscope (Leica, DMLS, Vienna, Austria).

### Lipopolysaccharide (LPS)-induced mouse calvarial bone erosion model

We constructed a LPS-stimulated bone destruction model in mouse calvaria based on our previous study 10. Healthy (age, 6-8 weeks) C57/BL6 mice were randomly divided into five groups (*n* = 6): (1) PBS control, (2) LPS, (3) LPS+ISL, (4) LPS+MSNs, and (5) LPS+MSNs-ISL. Under mild anesthesia, mice were subcutaneously injected with PBS or LPS (10 mg/kg body weight) at the midline of the calvarial sagittal suture for 7 days each. Then, the corresponding groups of mice were subcutaneously injected with (1) PBS, (2) PBS, (3) ISL (3.125 mg/kg body weight), (4) MSNs (50 mg/kg body weight), and (5) MSNs-ISL (50 mg/kg body weight) at 1 day before LPS injection and 30 min prior to the daily injection of LPS once in every 2 days. In the *in vivo* experiments, the ISL concentration was similar to that of ISL-encapsulated in the MSNs-ISL group, which was determined according to the procedure for calculating the loading and release efficacies. Following the final LPS injection, mice were sacrificed, and their calvaria was collected and fixed in 4% paraformaldehyde for 2 days.

### Micro-CT, HE, and TRAP staining

We constructed the 3D calvaria model using micro-CT (SkyScan1176, Burker Micro-CT, Belgium) at a resolution of 9 μm and analyzed the bone microstructure using the associated software. X-ray images of calvarial bone were obtained with In-Vivo DXS PRO (Bruker Corporation, Massachusetts, USA). For staining analysis, the calvaria were decalcified at room temperature in 10% EDTA for 14 days, embedded in paraffin, and then sectioned into 5 μm slices. The sections were stained with HE and TRAP to reveal bone erosion, and the osteoblasts and osteoclasts were quantified.

### Immunohistochemistry

After dewaxing, the sections were treated with gastric enzyme (MXB-BIO, Fuzhou, China) for 30 min. Immunohistochemistry was conducted using an UltraSensitive^TM^ SP (Mouse) IHC Kit (MXB-BIO, Fuzhou, China) according to the manufacturer's protocol. The sections were incubated overnight with a primary tumor necrosis factor-α (TNF-α) antibody (Santa Cruz Biotechnology, CA) at 4 °C. After processing using the kit, the sections were subjected to a chromogenic reaction with diaminobenzidine and the nuclei were labelled with hematoxylin. The intensity of immunohistochemical staining was then quantified.

### Double-immunofluorescence labelling

Sections were dewaxed and treated with gastric enzyme as described above, blocked for 1 h with normal goat serum (ZSGB-BIO, Beijing, China) at 37 °C, and then incubated overnight with primary cathepsin K, NFATc1, and c-Fos antibodies (Santa Cruz Biotechnology, CA) at 4 °C. The sections were washed, incubated with either Dylight 594-conjugated goat anti-mouse IgG (Abbkine, California, USA) or Dylight 594-conjugated goat anti-rabbit IgG (Abbkine, California, USA) at 37 °C for 1 h, and then stained with FITC-labeled phalloidin (Sigma, St. Louis) for 40 min. Afterward, the sections were infiltrated with mounting medium containing DAPI (nuclear dye, ZSGB-BIO, Beijing, China), and immunofluorescence staining intensity was quantified.

### Statistical analysis

Data are presented as mean ± standard deviation (SD). The statistical significance of the results was analyzed by one-way ANOVA followed by Student-Newman-Keul's test using SPSS 13.0 software (SPSS Inc, Chicago, IL, USA). Values were compared using multiple comparisons, and a *P* value < 0.05 was considered statistically significant.

## Results

### Synthesis and characterization of MSNs and MSNs-ISL

**Figure 1:**
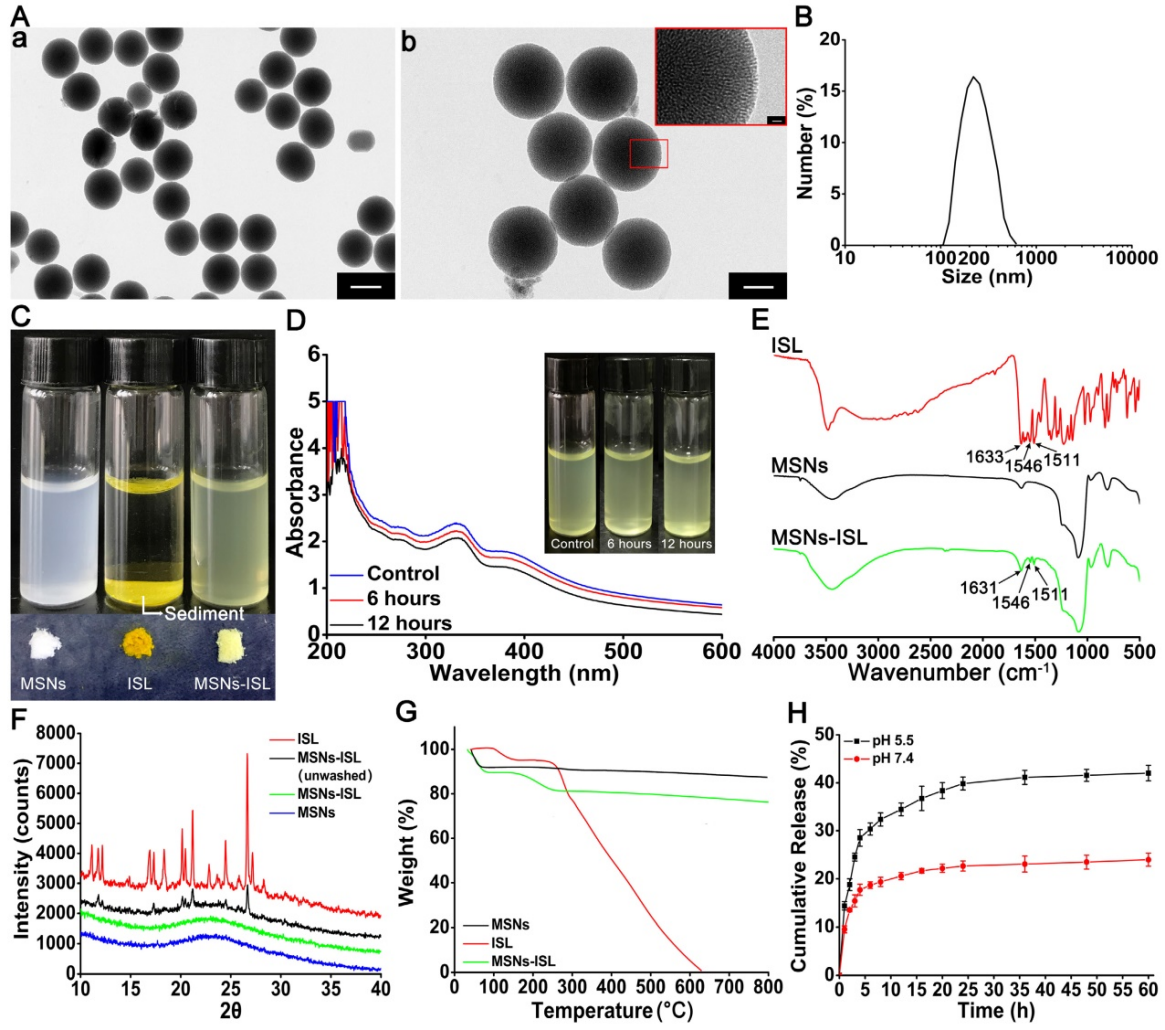
Characterization of MSNs and ISL loaded into and released from MSNs. (A) The TEM images of MSNs. (a) Scale bar = 200 nm. (b) Scale bar = 100 nm. (B) Average size of MSNs. (C) Images of MSNs, ISL and MSNs-ISL (1 mg) that were ultrasonically dispersed in the aqueous solution (2 mL) and their powders. (D) Images of MSNs-ISL that were uniformly dispersed in the aqueous solution (2 mL; control) or were subjected to stand still for 6 or 12 h. At the indicated time point, the upper half of the liquid (1 mL) was taken from the container and the UV-Vis spectra of MSNs were presented. (E) The FTIR spectra of ISL (red), MSNs (black) and MSNs-ISL (green). (F) The XRD patterns of ISL (red), un washed MSNs-ISL (black), MSNs-ISL (green), MSNs (blue). (G) The TGA thermograms of ISL (red), MSNs (black), and MSNs-ISL (green). (H) The cumulative percentage release of ISL from MSNs. The release was assessed using the UV-Vis method (*n* = 6).

TEM images indicated the uniform size of the synthesized MSNs (Figure 1A-a) and their radial nanochannel structure (Figure 1A-b). The average particle diameter of the MSNs was 224.36 ± 2.62 nm (Figure 1B), the size of which was selected both to minimize the potential inflammatory response and achieve anti-osteoclastogenesis effects 26, 27. MSNs-ISL were confirmed to have successfully encapsulated ISL by comparing their color with that of MSNs and ISL, and they were connected by electrostatic interactions between the hydrogen bonds (silanol groups) and the carboxyl group 28. MSNs and MSNs-ISL were uniformly dispersed by ultrasound in aqueous solution, whereas ISL was precipitated (Figure 1C). After the aqueous solution of MSNs-ISL was subjected to standing for the indicated time, the solution showed stability with slow aggregation (Figure 1D), likely because of hydrogen-bonding interactions between the surface silanol groups 29. The surface area (BET) and the pore diameter of MSNs was 960.3 m^2^ g^-1^ and 4.57 nm, respectively, whereas those of MSNs-ISL was 807.2 m^2^ g^-1^ and 4.15 nm, respectively (Table S1). The pore size provided sufficient vacancies for the entry of small molecule flavonoids into the interior channel 20. After ISL loading, the zeta potential in the aqueous solution decreased from -30.4 mV to -38.2 mV, whereas the absolute value increased (Table S1).

FTIR spectra were utilized to confirm ISL encapsulation; the characteristic infrared absorption frequencies of ISL and MSNs were compared with those of MSNs-ISL in the spectral range of 550-4000 cm^-1^ (Figure 1E). ISL has a vibration peak representing the enolic hydroxyl group at 3480 cm^-1^. The peak width of MSNs-ISL increased in comparison with that of the silicon hydroxyl peak of the MSNs. This finding indicates a possible enhancement in the intermolecular hydrogen bonding effect 30. The characteristic peak of MSNs-ISL at 1631 cm^-1^ and those at 1546 and 1511 cm^-1^ were respectively ascribed to C=O bonds and the aromatic nucleus of ISL 31. We analyzed the XRD patterns of ISL crystals, MSNs, unwashed MSNs-ISL and MSNs-ISL (Figure 1F). Drug loading on the surface of unwashed MSNs-ISL induced diffraction peaks characteristic of ISL crystals. However, these peaks disappeared after rinsing, thereby indicating that the ISL crystals had been removed from the surface of MSNs and that the ISL molecules in the pores presented a molecular amorphous state 32.

UV-Vis analysis revealed that the EE of ISL was 21.14% ± 1.04%. TGA analysis demonstrated that the total weight losses of MSNs-ISL and MSNs after heating are 24.59% ± 1.12% and 8.83% ± 0.47%, respectively, and the LE of ISL was 15.76% ± 1.02% (Figure 1G). MSNs-ISL were dispersed in the dissolution medium for 60 h to determine whether the ISL release is pH sensitive (Figure 1F). The ISL release system exhibited a significant reduction in the initial burst in 24 h at pH 5.5 or 16 h at pH 4.7. At 60 h, ISL release at pH 5.5 increased to 42.02% ± 1.63%, which is higher than that at pH 7.4 (23.97% ± 1.35%) (Figure 1H). ISL exhibited an explosive release at the beginning, which was suitable as it plays an important role in the early stage of anti-osteoclastogenesis 8. The calculations indicated that 16 and 64 μg of MSNs-ISL release approximately 1 and 4 μg of ISL, respectively.

### Effects of MSNs and MSNs-ISL on RANKL-stimulated osteoclastogenesis from BMMs

**Figure 2:**
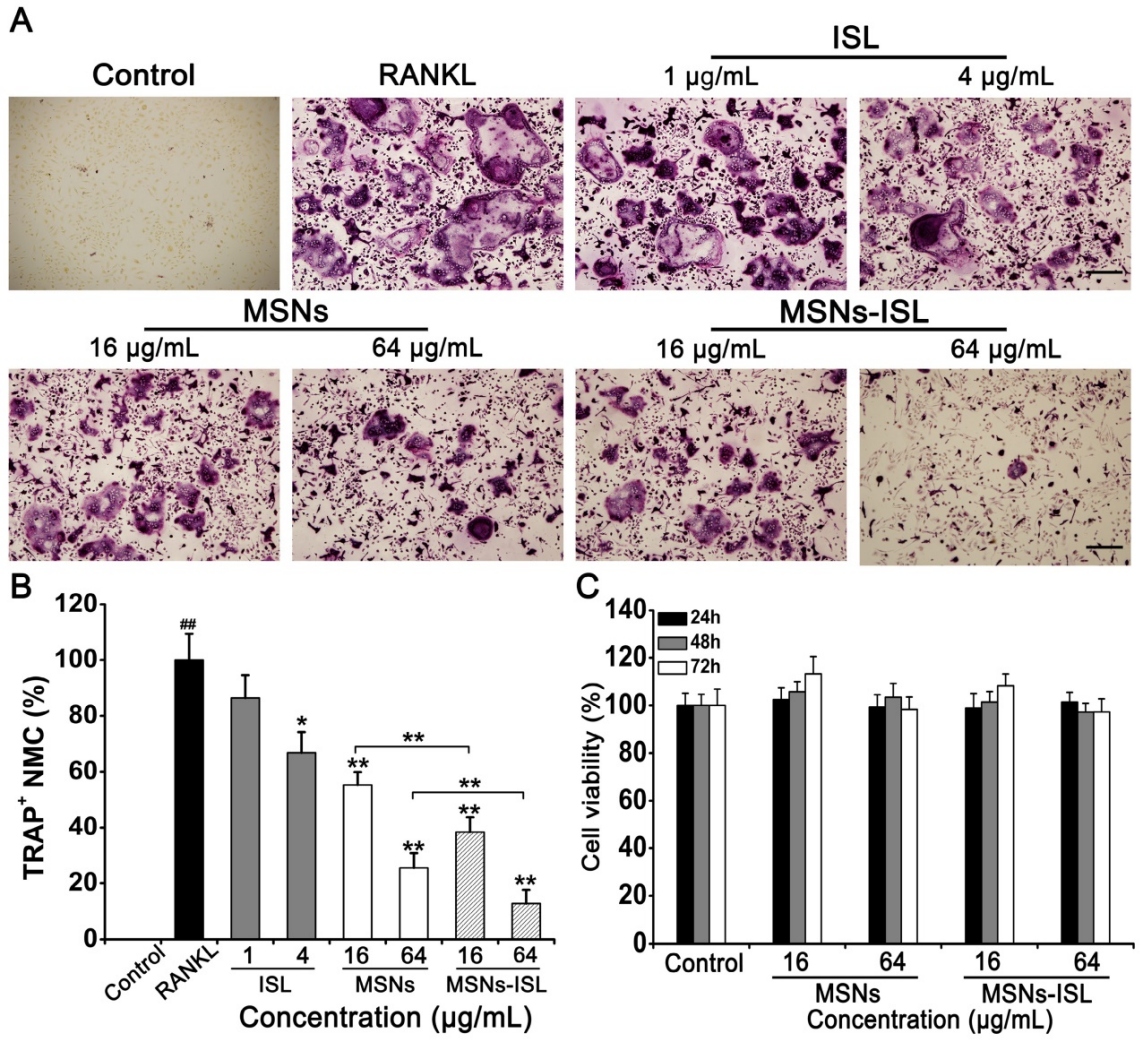
MSNs and MSNs-ISL suppressed RANKL-stimulated osteoclastogenesis from BMMs without cytotoxicity. Cells were incubated with 30 ng/ml M-CSF and 50 ng/mL RANKL plus various doses of either ISL, MSNs, or MSNs-ISL for 5 days. (A) Mature osteoclasts were labeled with TRAP staining. Scale bar = 200 μm. (B) TRAP-positive multinucleated cells (TRAP^+^ MNCs) with multiple nuclei (3 or more) were called osteoclasts and quantitated as the percentage of positive cells in the RANKL group. ^##^ *P* < 0.01 RANKL group vs. control group; ** *P* < 0.01, * *P* < 0.05 vs. RANKL group or MSNs group vs. MSNs-ISL group (*n* = 3). (C) Cells were incubated with various doses of MSNs and MSNs-ISL for 24, 48, and 72 h. Cell viability was determined using the Cell Counting Kit-8 assay (*n* = 3).

Mature osteoclasts (TRAP-positive cells) were studied through RANKL stimulation 33. MSNs and MSNs-ISL significantly reduced the quantity and size of osteoclasts in a dose-dependent manner, ISL exhibited osteoclast inhibition at a dose of 4 μg/mL only (Figure 2A). The highest dose of ISL used in our study (4 μg/mL) is within the range from 0 to 20 μM (5.125 μg/mL) which does not exert obvious cytotoxicity in BMMs 8, 10. Statistical results showed that fewer mature osteoclasts were obtained under MSNs-ISL stimulation than under MSNs stimulation, indicating the additive role of ISL and MSNs (Figure 2B). Good biocompatibility is one of the major advantages of MSNs as a drug delivery vehicle 15. We determined that our synthesized MSNs show no evident cytotoxicity to BMMs and MC3T3-E1 (Figure S1). To verify whether blockade of osteoclastogenesis by MSNs-ISL is a result of the potential toxicity of these nanoparticles, we assayed the cytotoxicity of the indicated concentrations of MSNs and MSNs-ISL at different time points. As revealed in Figure 2C, after incubation for 24, 48, and 72h, neither MSNs nor MSNs-ISL exhibited any cytotoxicity to BMMs. These results suggest that the effects of MSNs and MSNs-ISL on osteoclastogenesis are not attributed to cell proliferation changes.

### Effects of MSNs and MSNs-ISL on RANKL-stimulated F-actin rings and bone resorption pits formation

**Figure 3:**
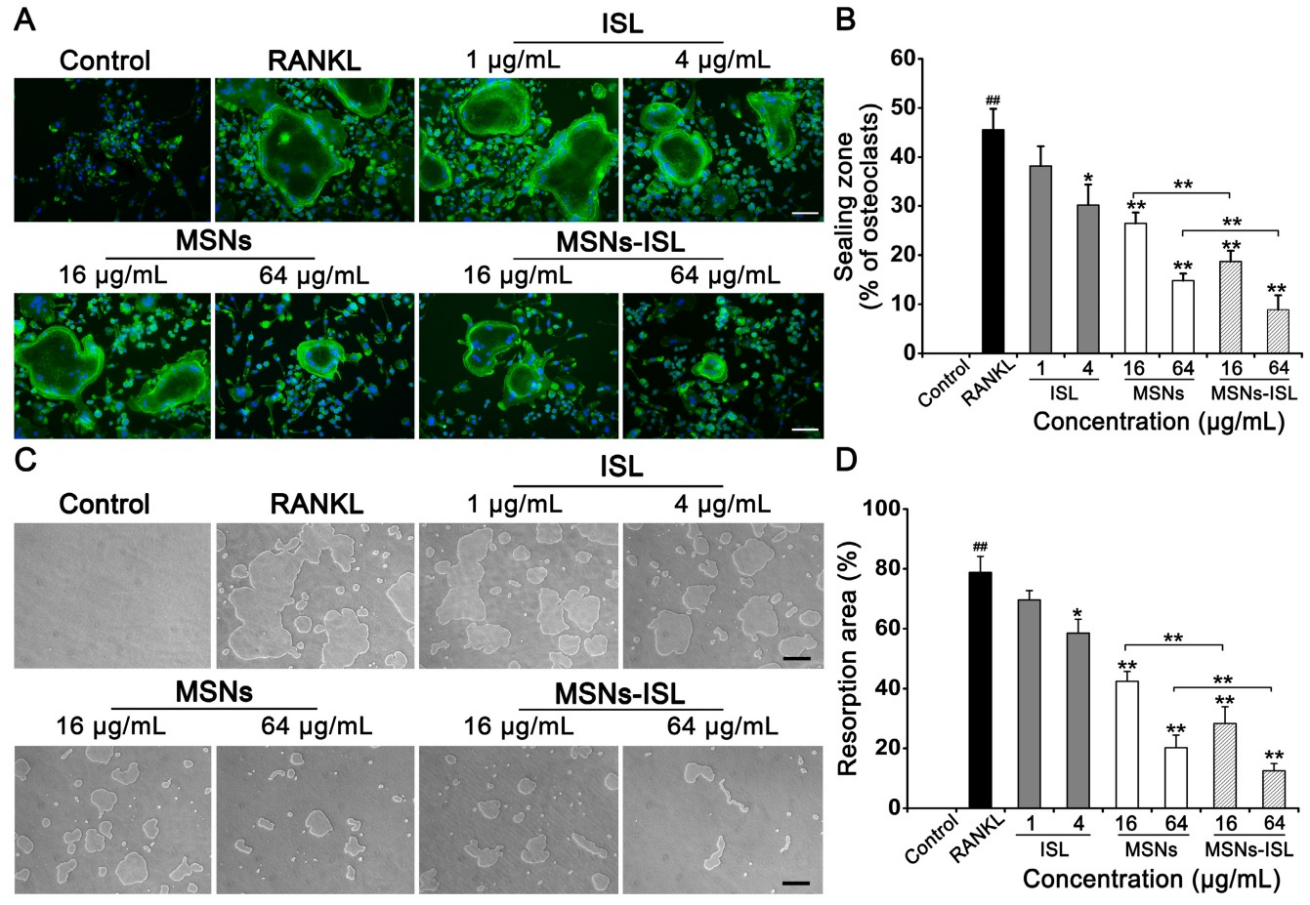
MSNs and MSNs-ISL inhibited RANKL-stimulated F-actin rings and bone resorption pits formation. (A) BMMs were incubated with 50 ng/mL RANKL plus various doses of either ISL, MSNs or MSNs-ISL for 5 days. F-actin rings were observed by phalloidin staining. Scale bar = 100 μm. (B) Osteoclasts with formation of the F-actin sealing zone were quantitated as percentages of the total number of osteoclasts. ^##^ *P* < 0.01 RANKL group vs. control group; ** *P* < 0.01, * *P* < 0.05 vs. RANKL group or MSNs group vs. MSNs-ISL group (*n* = 3). (C) BMMs were seeded into a Corning osteo assay surface multiple well plate with 50 ng/mL RANKL plus various ISL, MSNs and MSNs-ISL doses and were removed after 5 days. The bone resorption pits were then observed. Scale bar = 200 μm. (D) Resorption area was quantitated as the percentage of the total well area.^ ##^ *P* < 0.01 RANKL group vs. control group; ** *P* < 0.01, * *P* < 0.05 vs. RANKL group or MSNs group vs. MSNs-ISL group (*n* = 3).

F-actin rings, one of the components of sealing zones, can characterize the bone resorption capacity of induced osteoclast-like cells 34. Therefore, we analyzed whether MSNs and MSNs-ISL inhibit the formation of RANKL-stimulated F-actin rings in BMMs. We observed that the sealing zone decreases in a dose-dependent manner after MSNs and MSNs-ISL treatment, and that ISL promotes limited alteration on the sealing zone (Figure 3A). Compared with the MSNs group, the MSNs-ISL group presented less osteoclast formation and a smaller area of F-actin rings (Figure 3B). To explore the inhibitory activity of MSNs and MSNs-ISL on osteoclast bone resorption, we cultured BMMs on a Corning osteo assay surface multiple well plate, which provides a smooth surface for RANKL-stimulated osteoclasts and facilitates the formation of clear bone resorption pits 35. While the MSNs and MSNs-ISL treatments significantly reduced the areas of bone absorption pits, the ISL group showed several resorption pits with large areas resembling those in the control group (Figure 3C). Agreeing with the results of osteoclasts formation, the MSNs-ISL group showed fewer resorption pits than in the MSNs group (Figure 3D).

### MSNs inhibited RANKL-stimulated osteoclastogenesis at an early stage

**Figure 4:**
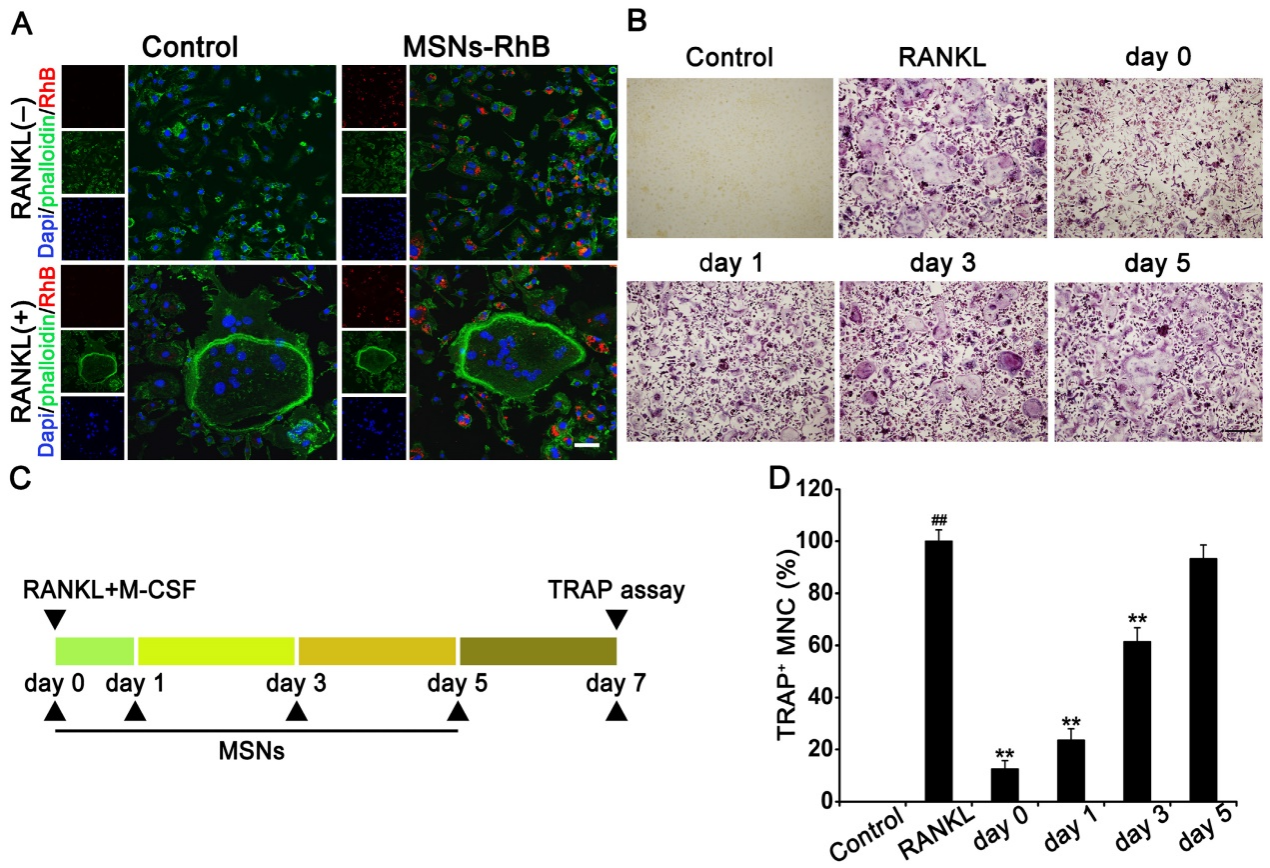
MSNs inhibited RANKL-stimulated osteoclastogenesis at an early stage. (A) BMMs or osteoclasts were incubated with 100 μg/mL of MSNs-RhB (red) for 12h. After fixation, FITC-conjugated phalloidin (green) and DAPI (blue) staining was performed. Cells were observed and imaged using a confocal laser scanning microscope. Scale bar = 50 μm. (*n* = 3). (B) BMMs were incubated with 30 ng/ml M-CSF and 50 ng/ml RANKL plus 100 μg/mL MSNs addition at the indicated time points (day 0 to day 5). Cells were fixed and TRAP staining was performed. Scale bar = 200 μm. (C) Schedule of experiments that determine the stage at which MSNs blocked osteoclastogenesis. (D) TRAP^+^ MNCs with multiple nuclei (3 or more) were quantitated as the percentage of positive cells in the RANKL group. ^##^* P* < 0.01 vs. control group. ** *P* < 0.01 vs. RANKL group (*n* = 3).

Osteoclastogenesis is a multi-step process that involves cell proliferation, commitment, and fusion 1. RhB is a fluorescent model drug that is considered suitable to observe the cellular uptake of nanoparticles 25. To confirm whether the osteoclast precursors or osteoclasts could phagocytose MSNs, MSNs-RhB were added to BMMs and mature osteoclasts. CLSM revealed that substantial intercellular uptake occurs both in osteoclast precursors and osteoclasts (Figure 4A). We previously demonstrated that ISL inhibits RANKL-stimulated osteoclastogenesis at an early stage 8. To determine the stage at which MSNs block osteoclastogenesis, nanoparticles were added to osteoclast differentiation cultures beginning on day 0 up to day 5 (Figures 4B and 4C). Statistical results showed that MSNs addition on the first day significantly reduced the mature osteoclasts, whereas the intercellular uptake of MSNs at day 5 was invalid for osteoclastogenesis inhibition (Figure 4D). We thus determined that MSNs inhibit the early cellular processes of osteoclastogenesis.

### Effect of MSNs and MSNs-ISL on RANKL-initiated mRNA expression of osteoclast-related genes and transcription factors

**Figure 5:**
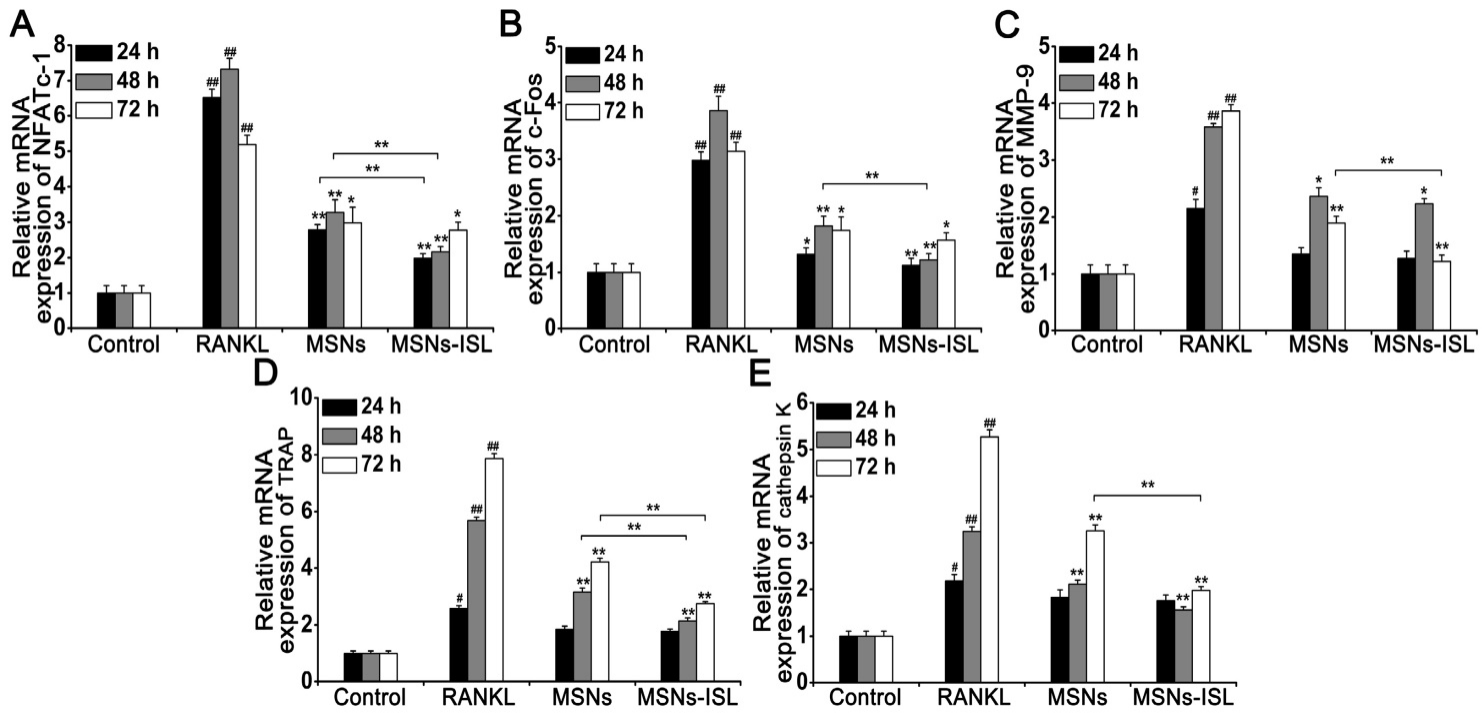
MSNs and MSNs-ISL suppressed the RANKL-initiated mRNA expression levels of osteoclast-related genes and transcription factors. (A-E) BMMs were incubated with 50 ng/mL RANKL plus 64 μg/mL of MSNs or MSNs-ISL for 24, 48, or 72 h. The relative mRNA expression levels of NFATc1 (A), c-Fos (B), MMP-9 (C), TRAP (D), and cathepsin K (E) are presented as compared with the relative mRNA expression levels of GAPDH. ^##^* P* < 0.01, ^#^* P* < 0.05 vs. control group; ** P < 0.01, * P < 0.05 vs. RANKL group or MSNs group vs. MSNs-ISL group (*n* = 3).

Osteoclast differentiation is suggested to be related to RANKL-mediated increases in specific gene expression, including that of NFATc1, c-Fos, matrix metalloprotein-9 (MMP-9), TRAP, and cathepsin K 1. qRT-PCR revealed a significant increase in the associated expression of these genes in BMMs through RANKL stimulation. Treatment with MSNs and MSNs-ISL significantly downregulated the related mRNA expression at various time points. Both treatments reduced NFATc1 mRNA expression levels at all time points, with statistically significant differences observed between the two groups at 24 and 48 h (Figure 5A). The mRNA expression of c-Fos was also significantly decreased at all time points; however, the difference between MSNs and MSNs-ISL was statistically significant only at 48 h (Figure 5B). The inhibited mRNA expression of MMP-9, TRAP, and cathepsin K changed significantly only after 48 and 72 h of treatment with MSNs and MSNs-ISL. The two groups showed statistically significant differences in the mRNA expression of MMP-9 and cathepsin K at 72 h and in the mRNA expression of TRAP at 48 and 72h (Figure 5C-5E). Taken together, MSNs could suppress RANKL-induced mRNA expression of these genes, and MSNs-ISL show stronger effects than MSNs.

### Effect of MSNs and MSNs-ISL on osteoclast-associated signaling pathways

**Figure 6:**
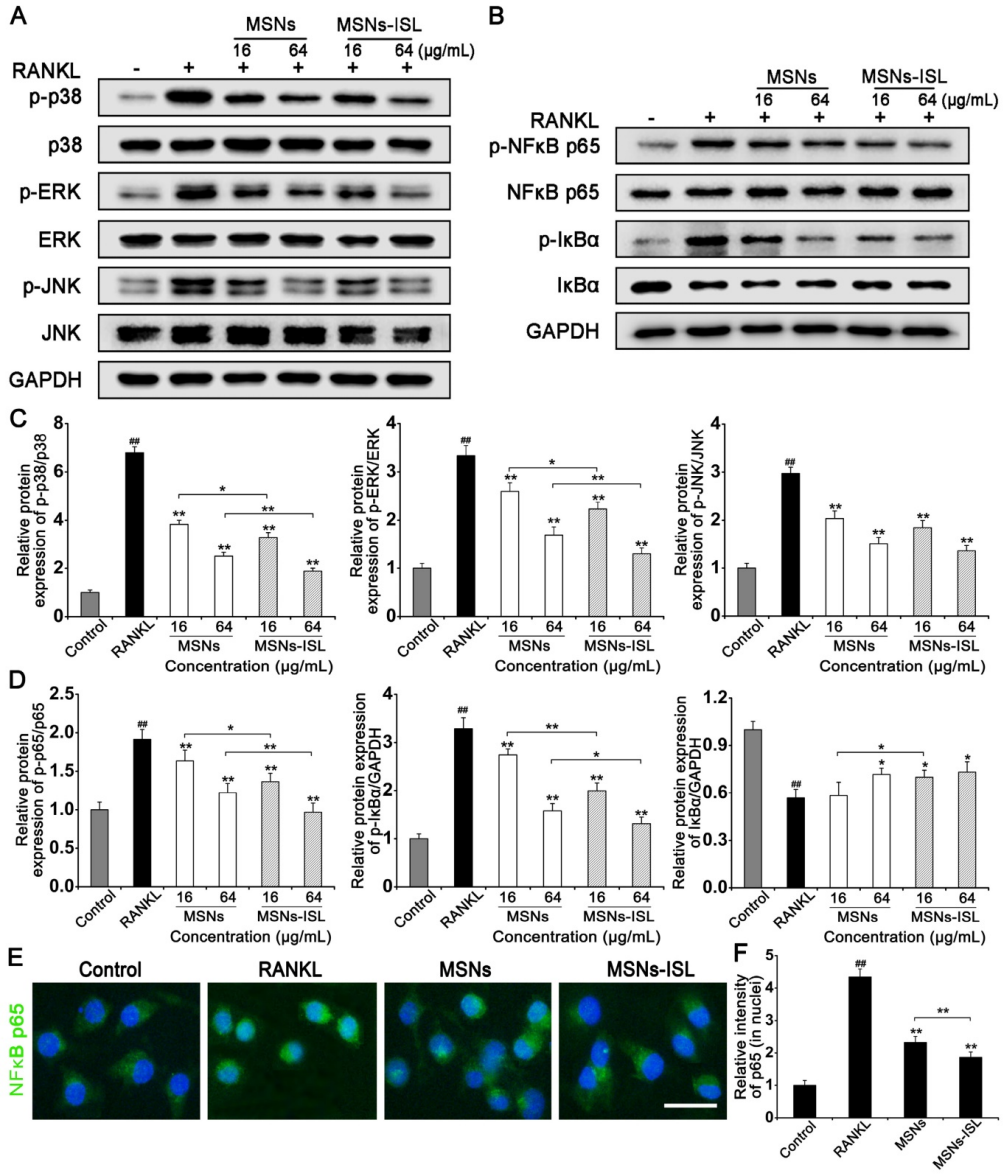
Effect of MSNs and MSNs-ISL on RANKL-initiated activation of NF-κB and MAPK signaling pathways. (A) BMMs were incubated with 50 ng/mL RANKL plus various doses of either MSNs or MSNs-ISL for 30 min. Protein levels in the MAPK pathways were analyzed via western blot analysis (*n* = 3). (B) BMMs were incubated with 50 ng/mL RANKL plus various doses of either MSNs or MSNs-ISL for 30 min. Protein levels in the NF-κB pathway were analyzed via western blot analysis (*n* = 3). (C) The relative protein expression levels of p-p38/p38, p-ERK/ERK, and p-JNK/JNK.^ ##^* P* < 0.01 vs. control group; ** P < 0.01, * P < 0.05 vs. RANKL group or MSNs group vs. MSNs-ISL group. (D) The relative protein expression of p-p65/p65, p-IκBα/GAPDH and IκBα/GAPDH.^ ##^* P* < 0.01 vs. control group; ** P < 0.01, * P < 0.05 vs. RANKL group or MSNs group vs. MSNs-ISL group. (E) BMMs were cultured with 50 ng/mL RANKL plus 64 μg/mL MSNs or MSNs-ISL for 12 h. The location of NF-κB p65 (green) was identified though an immunofluorescence assay. The nuclei (blue) were marked with 4',6-diamidino-2-phenylindole (DAPI). Scale bar = 50 μm. (F) The intensity of NF-κB p65 (in nuclei) staining was quantitated. ^##^ *P* < 0.01 vs. control group; ** *P* < 0.01 vs. RANKL group or MSNs group vs. MSNs-ISL group (*n* = 3).

Nonporous silica nanoparticles approximately 200 nm in size could suppress osteoclastogenesis by decreasing receptor activator of nuclear factor-κB (RANK) together with NFATc1 gene expression 26. Our original study confirmed that ISL blocks RANK-triggered association with TRAF6, and inhibits activation of the NF-κB and mitogen-activated protein kinases (MAPKs)/activator protein (AP)-1 pathways 8. Thus, we explored the mechanisms underlying the inhibition of osteoclastogenesis by stimulation with MSNs and MSNs-ISL. MSNs and MSNs-ISL diminished the phosphorylation of p38 MAPK, extracellular signal-regulated protein kinase ERK 1/2, and JNK (Figure 6A); however, the same concentrations, did not induce statistically significant differences between two groups in the phosphorylation of JNK (Figure 6C). MSNs and MSNs-ISL also reduced RANKL-mediated IκBα phosphorylation and degradation, as well as subsequent NF-κB p65 phosphorylation, in a dose-dependent manner (Figure 6B). The MSNs-ISL group presented more evident inhibitory effects on NF-κB activation than the MSNs group (Figure 6D). To verify the western blot results, we performed immunofluorescence staining analysis. As shown in Figure 5E, NF-κB p65 was mainly located in the cytoplasm in the control group. After RANKL stimulation, the cells showed NF-κB p65 activation and translocated to the nuclei. MSNs and MSNs-ISL could reduce NF-κB p65 nuclear translocation mediated by RANKL, and the difference between groups was statistically significant (Figure 6F).

**Figure 7:**
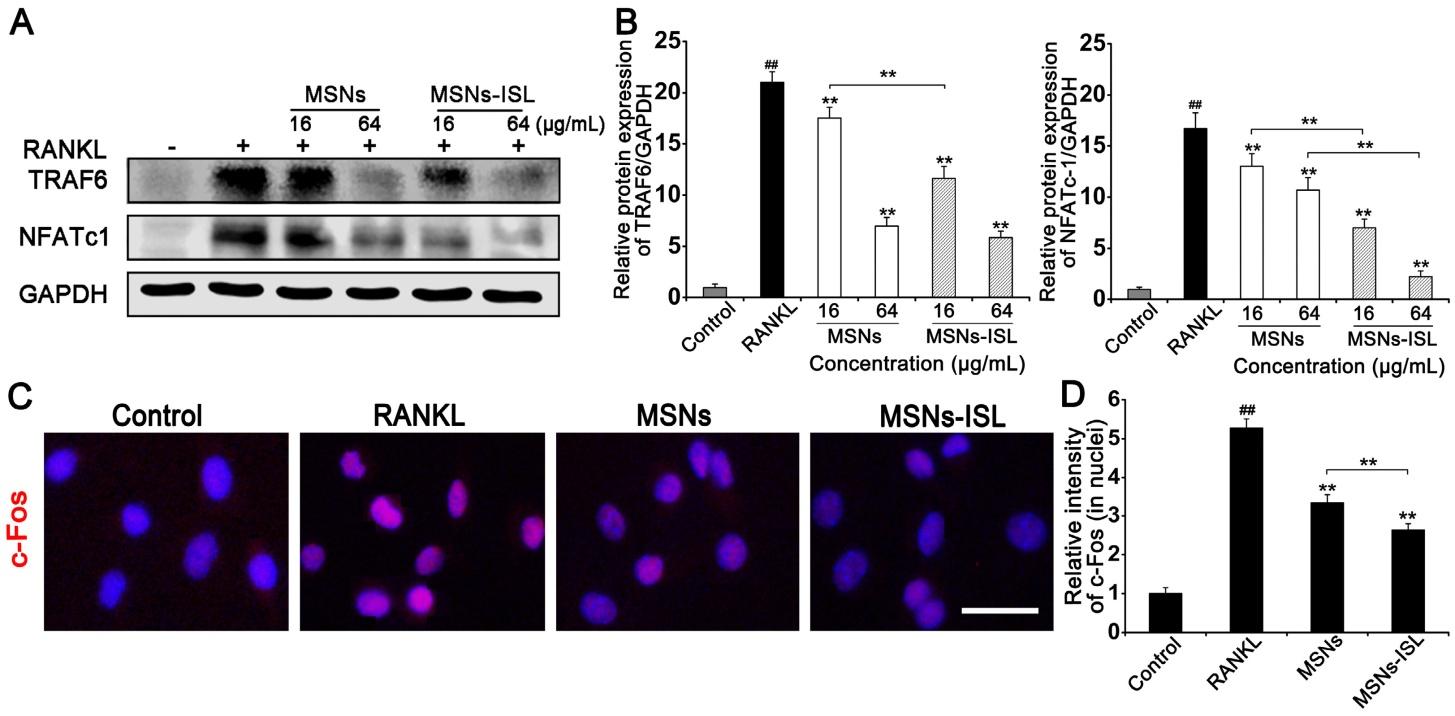
Effect of MSNs and MSNs-ISL on TRAF6-coordinated osteoclastogenic transcriptional factors. (A) BMMs were cultured with 50 ng/mL RANKL plus various concentrations of either MSNs or MSNs-ISL for 24 h. Western blot analysis was carried out to analyze the protein levels of TRAF6 and NFATc1. (B) The relative protein expression levels of TRAF6/GAPDH and NFATc1/GAPDH.^ ##^* P* < 0.01 vs. control group; ** P < 0.01, * P < 0.05 vs. RANKL group or MSNs group vs. MSNs-ISL group (*n* = 3). (C) BMMs were cultured with 50 ng/mL RANKL plus 64 μg/mL MSNs or MSNs-ISL for 24 h. The location of c-Fos (red) was identified though an immunofluorescence assay. The nuclei (blue) were marked with DAPI. Scale bar = 50 μm. (D) The intensity of c-Fos (in nuclei) staining was quantitated.^ ##^* P* < 0.01 vs. control group; ** P < 0.01 vs. RANKL group or MSNs group vs. MSNs-ISL group (*n* = 3).

Activation of NFATc1 and TRAF6 was significantly reduced by MSNs and MSNs-ISL in a dose-dependent manner (Figure 7A); indeed, the latter almost completely eliminated the activation of NFATc1 (Figure 7B). Immunofluorescence staining analysis was performed to confirm the nuclear translocation of the AP-1 component c-Fos, which was inactive and mainly located in the cytoplasm in the absence of RANKL (Figure 7C). Evident nuclear translocation of c-Fos was observed after incubation with RANKL. However, MSNs and MSNs-ISL could attenuate c-Fos nuclear translocation, although the latter showed less translocation intensity than the former (Figure 7D).

### Effects of MSNs and MSNs-ISL on LPS-mediated calvarial bone destruction* in vivo*

**Figure 8:**
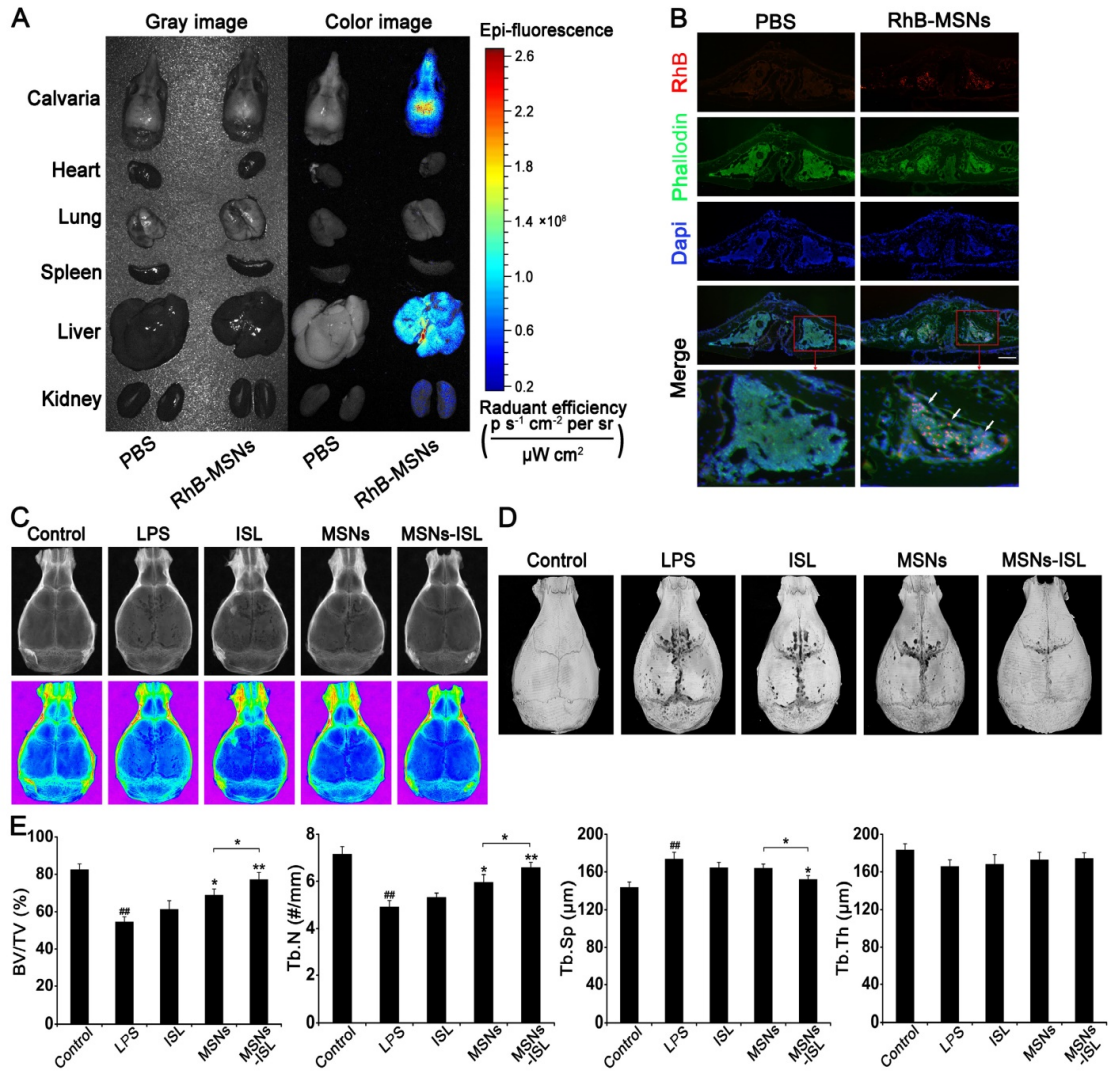
Tissue distribution of MSNs-RhB *in vivo* (A-B). Mice were subcutaneously injected with PBS or MSNs-RhB at the midline of the calvarial sagittal suture for 3 hours. (*n* = 6) (A) Representative images for the tissue distribution of MSNs in mice by biophotonic imaging. The fluorescence signals were analyzed in the isolated calvarial bone, hearts, lungs, spleens, livers, and kidneys of mice. (B) Representative fluorescence images of calvarial bone cryosections. The cytoskeleton and bone were labeled with phalloidin (green) and the nuclei were labeled with DAPI (blue). Scale bar = 200 μm. The white arrows indicate MSNs labeled with RhB (red). MSNs and MSNs-ISL prevented LPS-mediated calvarial bone erosion in mice (C-E). Mice were injected with either ISL (3.125 mg/kg body weight), MSNs (50 mg/kg body weight), or MSNs-ISL (50 mg/kg body weight), one day before LPS (10 mg/kg body weight) injection and 30 min before the daily injection of LPS, once every 2 days. After 7 days, the mice were sacrificed after the final LPS injection. (*n* = 6) (C) X-ray images. (D) Micro-CT 3D reconstruction images. (E) Morphometric indices: BV/TV (a), Tb.N (b), Tb.Th (c), and Tb.Sp (d) were analyzed by micro-CT. ^##^* P* < 0.01 vs. control group; ** P < 0.01, * P < 0.05 vs. LPS group or MSNs group vs. MSNs-ISL group.

We revealed in previous experiments that ISL can inhibit LPS-stimulated bone destruction both locally and systemically *in vivo* 8, 10. Nonporous silica nanoparticles can also increase the bone mineral density in mice 36. To verify whether MSNs and MSNs-ISL have similar effects, we constructed a mouse calvarial model. Using RhB as a fluorescent model drug, we observed that some MSNs-RhB is distributed in the calvarial bone after injection for 3 hours; the rest enters the liver and kidney metabolism (Figure 8A). Observation of calvarial cryosections revealed that MSNs-RhB are taken up by cells (Figure 8B). We were thus able to demonstrate the feasibility of local application of MSNs to the mice calvarial model. The X-ray images in Figure 8C show the bone resorption results. Micro-CT scanning and 3D reconstruction depicted a large area of crater-like bone destruction on the surface of LPS-injected calvarias. Compared with the ISL group, reduced bone absorption area was evident in the MSNs and MSNs-ISL groups (Figure 8D). The MSNs and MSNs-ISL reversed the decreased bone volume/total volume (BV/TV) and trabecular bone number (Tb.N), along with increased trabecular separation (Tb.Sp) under LPS stimulation with a significant difference between the two groups. Trabecular thickness (Tb.Th) was high in the MSNs-ISL group (*P* > 0.05) but insignificantly different among the five groups (Figure 8E).

**Figure 9:**
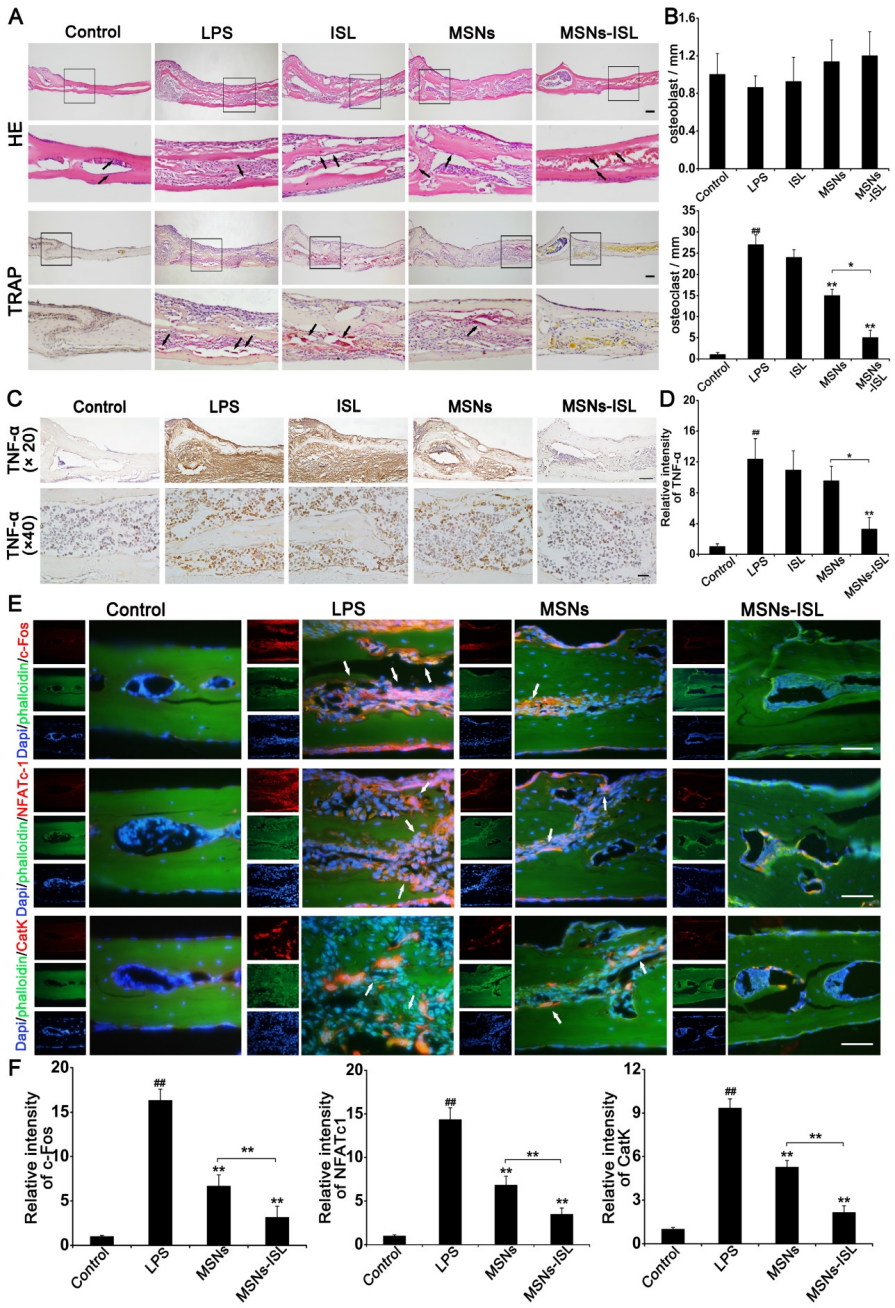
MSNs and MSNs-ISL protected against inflammatory mice calvarial bone resorption. Histological analysis was conducted on the calvaria sections. (A) Histological staining was performed on the mice calvarial bone sections subjected to HE and TRAP staining. Scale bar = 200 μm. The black arrows indicate osteoblasts (HE staining) and TRAP positive cells (TRAP staining). (B) Osteoblasts and TRAP positive cells were quantified. ^##^* P* < 0.01 vs. control group; ** P < 0.01, * P < 0.05 vs. LPS group or MSNs group vs. MSNs-ISL group. (C) Immunohistochemical images for the distribution of TNF-α. Hematoxylin was used to labeled the nuclei (blue). Scale bar = 100 μm (× 20). Scale bar = 20 μm (× 40). (D) The relative intensities of TNF-α were quantitated. ^##^ *P* < 0.01 vs. control group; ** *P* < 0.01 vs. LPS group or MSNs group vs. MSNs-ISL group. (E) Representative immunofluorescence images for c-Fos, NFATc1, and cathepsin K (CatK) expression. The cytoskeleton and bone were labeled with phalloidin (green) and the nuclei (blue) were labeled with DAPI. Scale bars = 50 μm. The white arrows indicate positive expression cells. (F) The relative intensities of c-Fos, NFATc1 and CatK were quantitated. ^##^* P* < 0.01 vs. control group; ** P < 0.01 vs. RANKL group or MSNs group vs. MSNs-ISL group.

The results of HE and TRAP staining in histological analysis confirmed that MSNs and MSNs-ISL could inhibit the inflammation-associated interruption of calvarial bone continuity (Figure 9A). HE and TRAP staining were used to count and osteoclasts, respectively (Figure 9B). Th statistical results showed that the number of osteoblasts was not statistically different between the groups (*P* < 0.05). TRAP staining revealed a few osteoclasts existing in the bone tissues of the MSNs and MSNs-ISL groups; indeed, the MSNs-ISL group was almost completely bereft of TRAP-positive cells. In the immunohistochemistry experiments, LPS induced the production of TNF-α, a pro-inflammatory cytokine 37. The MSNs-ISL group almost completely reversed the LPS-elicited expression of TNF-α, whereas the MSNs group slightly decreased the expression of TNF-α by LPS but without significant differences (Figures 9C and 9D). In immunofluorescence experiments, c-Fos, NFATc1, and cathepsin K expression was significantly increased after LPS treatment but declined in the MSNs group and was almost completely eliminated in the MSNs-ISL group (Figures 9E and 9F).

## Discussion

Increased osteoclast number along with excessive bone resorption is involved in various inflammation‐induced lytic bone disorders, including osteomyelitis, septic arthritis, and periodontitis 2. Our previous studies, as well as those of others, demonstrate that the licorice-derived bioactive flavonoid ISL could be a promising anti-bone destructive agent worthy of further development 8-10. However, several disadvantages of ISL, including its poor solubility, short elimination half-life, and low bioavailability, severely limit its applications in biomedical and clinical fields 11. Novel nanotechnology and drug delivery systems offer new opportunities to address these problems 18-20. In the present study, we encapsulated ISL into synthesized MSNs and found that the resulting MSNs-ISL feature a large specific surface area, superior biocompatibility, and favorably allow acid-sensitive sustained drug release. Our findings show, for the first time, that compared with free ISL and MSNs alone, MSNs-ISL provide prominent protection against osteoclast-mediated bone loss *in vitro* and *in vivo*.

Nanoencapsulation has attracted wide research attention due to its many benefits that include easy handling, high stability, and biological availability, controllable release, and enhanced water solubility 12-14. In the present study, the MSNs encapsulation system could successfully improve the solubility of ISL since the molecules of the latter present a molecular amorphous state and no crystallization occurs inside the MSNs pores 32. A previous study determined that the absolute value of the negative zeta potential of nonporous silica nanoparticles is directly proportional to the degree of osteoclastogenesis inhibition 26. Thus, the decrease in zeta potential during ISL encapsulation could be a potential factor influencing the increased osteoclastogenesis suppression induced by MSNs-ISL.

Osteoclasts dissolve bone matrix by forming acidic sealing zones on bone, which allow proton secretion and activation of acid-dependent proteases for bone resorption 38. The pH of the inflammatory tissue microenvironment is lower than that of normal tissue, and endosomes phagocytizing nanoparticles show low pH 39. The drug-release experiment in our study demonstrated that ISL is highly and cumulatively released from MSNs-ISL in an acidic environment. Interestingly, a previous study showed that flavonoids are more stable in acidic environments than in alkaline ones due to the ionization of phenolic hydroxyl groups in the latter 40. Therefore, the sensitivity of acid-responsive MSNs-ISL may define their on-demand release characteristics in osteoclasts or inflammatory tissues. The sustained-release properties of MSNs-ISL would also be beneficial for their biological effects on osteoclasts.

Nonporous silica nanoparticles are considered bone bioresponsive and can significantly suppress osteoclast formation 26, 36. Nevertheless, the effects of the widely utilized form of MSNs on osteoclast formation and function remain unknown. Here, we demonstrated that MSNs suppressed RANKL-mediated osteoclast formation, decreased the size and number of the sealing zones, and reduced the osteolytic capacity of osteoclasts. The reorganization and aggregation of sealing zones is closely related to the functionality of osteoclasts in bone resorption 34. MSNs-ISL almost completely restrained osteoclast formation and function.

**Scheme 1:**
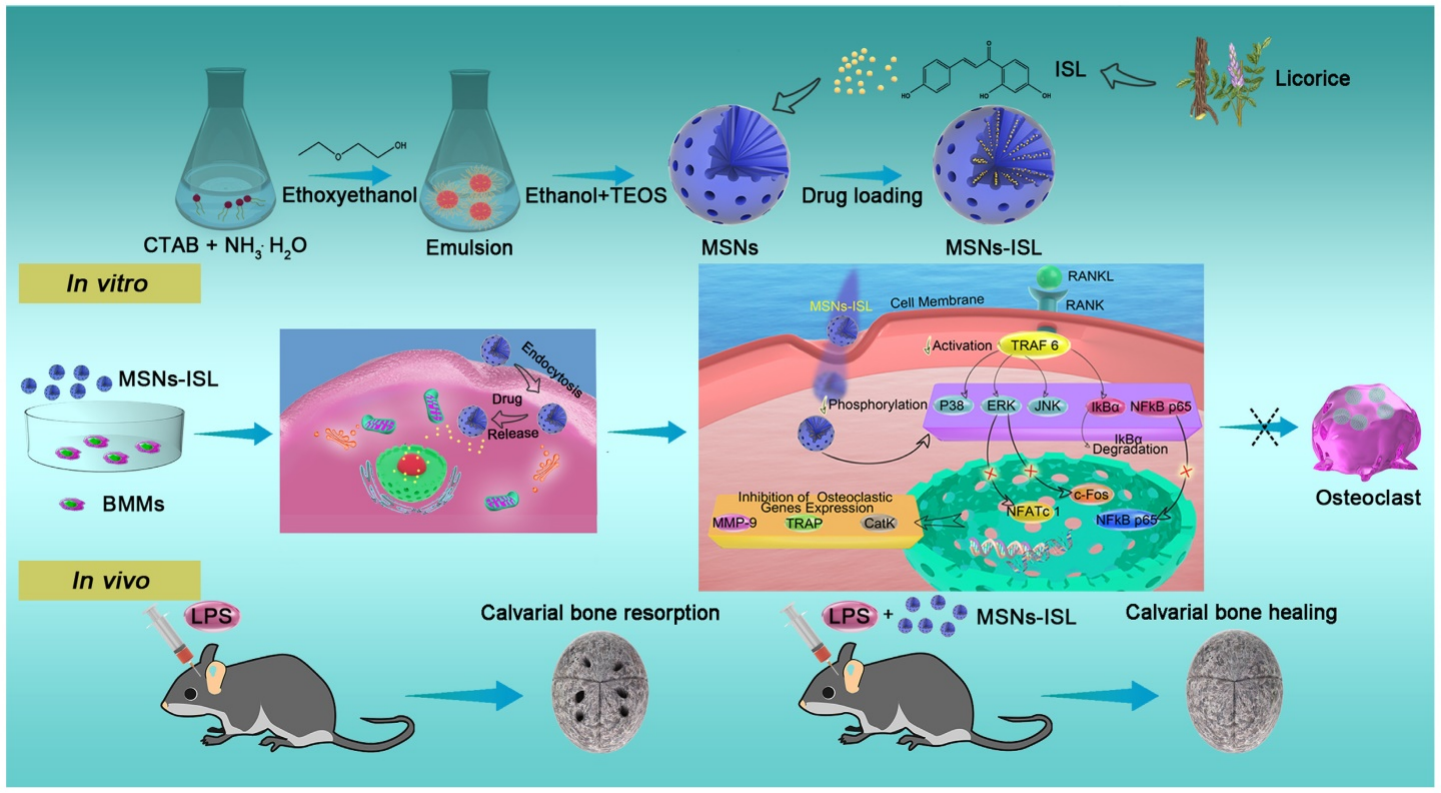
Scheme of the synthesis and biological effects of the natural product-based bone-bioresponsive nanoencapsulation system MSNs-ISL. During *in vitro* therapy, BMMs cannot be differentiated into activated osteoclasts due to phagocytosed MSNs-ISL. An integrated blueprint was constructed for the intracellular molecular mechanism of MSNs-ISL-stimulated anti-osteoclastogenesis. During *in vivo* therapy, MSNs-ISL promoted cavarial bone healing from LPS-mediated inflammatory bone resorption.

We further explored the mechanism underlying the phenomena described above. The importance of NF-κB and three other MAPKs namely, p38 MAPK, ERK 1/2, and JNK, in the RANKL/RANK pathway for osteoclastogenesis has been confirmed by genetic studies 41. Nonporous silica nanoparticles can inhibit activation of the RANKL-induced NF-κB pathway 36 and decrease the osteoclastogenesis-associated gene expression levels of RANK and NFATc1 26. However, the degrees of NF-κB and MAPK pathways activation in the presence of nonporous silica nanoparticles and their porous form MSNs is different 42, and the precise mechanism of RANKL-induced osteoclastogenesis under the stimulation by MSNs unexplored. We confirmed that MSNs could inhibit the activation of TRAF6, which is trimerized and activated after the binding of RANKL and its receptor RANK 41. Furthermore, MSNs restrain RANKL-stimulated MAPK and NF-κB pathway activities and decrease the expression levels of NFATc1 and c-Fos, two representative osteoclastogenic transcription factors induced upon the activation of these pathways 43. Compared with MSNs, MSNs-ISL could more remarkably suppress the activation of RANKL-stimulated osteoclast-related signaling pathways and significantly downgrade the expression of genes involved in osteoclast differentiation. MSNs-ISL almost completely revoked NFATc1 expression, consistent with our previous finding that NFATc1 is the main target of ISL 8. Our observations successfully constructed an integrated blueprint describing the intracellular molecular mechanism of MSNs-ISL in anti-osteoclastogenesis (Scheme 1).

LPS, a fundamental component in the cell membrane of gram-negative bacteria, is a critical pathogenic factor in inflammatory bone diseases 44. LPS can promote inflammatory bone destruction by increasing osteolytic resorption activity and extending the life span of mature osteoclasts 37. The established model of LPS-induced bone resorption in mouse calvaria is a suitable and clean system through which bone resorption initiated by infection and inflammation can be mimicked 45. A previous study confirmed that silicon (Si) shows anti-inflammatory properties toward RAW 264.7 cells by reducing LPS-induced TNF-α production 46. Macrophages notably release less pro-inflammatory cytokines under the stimulation of the more widely utilized forms with MSNs compared to their nonporous counterparts 42, and ISL relieves the LPS-induced systemic and local inflammatory bone resorption 8,10. Consistent with our results *in vitro*, pretreatment with MSNs and MSNs-ISL inhibited LPS-induced inflammatory bone destruction *in vivo*. Furthermore, MSNs-ISL showed more obvious inhibitory effects of osteoclastogenesis than MSNs due to the additive effects of ISL and MSNs. The protective effect of pretreatment with MSNs-ISL on the LPS-induced inhibition of osteoclastogenesis was confirmed by evaluating the effect of the nanoparticles on the expression of osteoclast-specific genes. MSNs-ISL revealed more intense suppression of c-Fos, NFATc1, and cathepsin K expression than MSNs. These signaling pathways are also critical for osteoclast differentiation 47. Cathepsin K, a major enzyme regulating bone and cartilage degradation, is abundantly expressed in osteoclasts as a marker protein for these cells 48.

## Conclusion

These findings show for the first time, that MSNs-ISL possess a large specific surface area and suitable porosity; they also favorably allow acid-sensitive sustained drug release. Thus, MSNs are superior osteoclast-bioresponsive nanocarriers with good biocompatibility. Compared with free ISL and MSNs, MSNs-ISL significantly and additively inhibited RANKL-induced osteoclast formation, decreased the area and quantity of sealing zones, and reduced the osteolytic capacity of osteoclasts *in vitro*. MSNs-ISL revealed their inhibitory effects by down-regulating TRAF6 expression, suppressing the activation of MAPKs and NF-κB, and subsequently decreased c-Fos and NFATc1 expression. Moreover, these nanoparticles can significantly attenuate osteoclast activity and relieve inflammation-associated calvarial bone destruction *in vivo*, which involves suppression of osteoclastogenetic c-Fos, NFATc1, and cathepsin K expression. The results of this study provide a proof of concept for the feasibility of applying MSNs-ISL as an effective natural product-based bone-bioresponsive nanoencapsulation system to prevent osteoclast-mediated bone loss.

## Supplementary Material

Supplementary methods, figures and tables.

## Abbreviations

AP: activator protein
BET: Brunauer-Emmett-Teller
BJH: Barrett-Joyner-Halenda
BMMs: bone marrow-derived macrophages
BV/TV: bone volume/total volume
CLSM: confocal laser scanning microscopy
CTAB: cetyltrimethyl ammonium bromide
DMSO: dimethyl sulfoxide
EE: encapsulation efficiency
ERK: extracellular signal-regulated protein kinase
FTIR: Fourier transform infrared spectroscopy
HE: hematoxylin-eosin
IκBα: inhibitor of κBα
ISL: Isoliquiritigenin
JNK: c-Jun N-terminal kinase
LE: loading efficiency
LPS: lipopolysaccharide
MAPKs: mitogen-activated protein kinases
M-CSF: macrophage colony-stimulating factor
MEM: modified Eagle's medium
micro-CT: micro-computed tomography
MMP-9: matrix metalloprotein-9
MSNs: mesoporous silica nanoparticles
MSNs-ISL: ISL-encapsulated MSNs
NFATc1: nuclear factor of activated T cells
qRT-PCR: quantitative real-time polymerase chain reaction
RANK: receptor activator of nuclear factor-κB
RANKL: receptor activator of nuclear factor-κB ligand
RhB: rhodamine B
Tb.N: trabecular bone number
Tb.Sp: trabecular separation
Tb.Th: trabecular thickness
TEOS: tetraethyl orthosilicate
TEM: transmission electron microscopy
TNF-α: tumor necrosis factor-α
TRAF6: tumor necrosis factor receptor-associated factor 6
TRAP: tartrate-resistant acid phosphatase
UV-Vis: ultraviolet-visible spectroscopy
XRD: X-ray diffraction
