# Supplementary material for: Licorice isoliquiritigenin-encapsulated mesoporous silica nanoparticles for osteoclast inhibition and bone loss prevention
Source: Theranostics. 2019 Jul 9;9(18):5183–99. doi: 10.7150/thno.33376 (PMC6691588; doi:10.7150/thno.33376)
Supplement: Supplementary file 1 — Supplementary methods, figures and tables. [file thnov09p5183s1.pdf]

Supplementary Information

Licorice isoliquritigenin-encapsulated mesoporous silica nanoparticles for osteoclast inhibition and bone loss therapy

Xiaoyue Sun[#], Jie Zhang[#], Zijun Wang, Bingqian Liu, Shenting Zhu, Lingxin Zhu*, Bin Peng*

The State Key Laboratory Breeding Base of Basic Science of Stomatology (Hubei-MOST) & Key Laboratory of Oral Biomedicine Ministry of Education, School & Hospital of Stomatology, Wuhan University, Wuhan 430079, China.

[#]Xiaoyue Sun and Jie Zhang contributed equally to this work.

*Corresponding authors: Lingxin Zhu: lingxin.zhu@whu.edu.cn; Bin Peng: phs301@whu.edu.cn

The State Key Laboratory Breeding Base of Basic Science of Stomatology (Hubei-MOST) & Key Laboratory of Oral Biomedicine Ministry of Education, School & Hospital of Stomatology, Wuhan University 237 Luoyu Road, Wuhan 430079, P.R.China. Tel: + 86 27 87161981; fax: +86 27 87873260.

Supplementary methods

Materials and reagents

TEOS and CTAB were purchased from Sigma-Aldrich (St. Louis, MO, USA). ISL was purchased from either Sigma-Aldrich (St. Louis, MO, USA; for cell experiments) or Mei5 Biotechnology Co. (Beijing, China; for drug loading and mice experiments). Recombinant murine soluble RANKL and M-CSF were purchased from Peprotech (London, UK). *Escherichia coli* serotype O55:B5 LPS was purchased from Sigma-Aldrich (St. Louis, MO, USA). Primary antibodies to p-p38, p38, p-ERK, ERK, GAPDH, p-NF-κB p65, NF-κB p65, IκBα, and p-IκBα were purchased from Cell Signaling Technology (Danvers, MA). The antibodies to p-JNK, JNK, TRAF6, NFATc1, c-Fos, cathepsin K and TNF-α were purchased from Santa Cruz Biotechnology (Santa Cruz, CA). Dylight 594- and 488-conjugated goat anti-mouse immunoglobulin G were purchased from Abbkine (California, USA). Fetal bovine serum, penicillin, streptomycin, and MEM were obtained from GIBCO BRL (Gaithersburg, MD, USA).

Cytotoxicity assay

BMMs and MC3T3-E1 were seeded respectively into 96-well plates at suitable densities and allowed to adhere overnight. Different MSNs concentrations (i.e., 1, 4, 12.5, 25, 50, 100 μg/mL) and 30 ng/mL M-CSF were added, and the cells were cultured for 24 h. Cell viability was evaluated using Cell Counting Kit-8 (CCK-8; Dojindo, Kumamoto, Japan) according to the manufacturer's protocol. The absorbance at 450 nm was assessed for all the wells using an ELISA microplate reader (Bio-Tek Instruments Inc., Winooski, VT, USA).

Cellular uptake of MSNs and MSNs-ISL

BMMs at a suitable density were incubated overnight in dishes; 64 μg/mL MSNs or MSNs supplements were added to the complete culture medium and cultured for 24 h. After washing with PBS, the cells were collected by centrifugation and fixed with 2.5% glutaraldehyde. The cellular uptake of the nanoparticles was observed using a transmission electron microscope (TEM; Hitachi, Japan) at an accelerating voltage of 30 kV and 80 kV, and was quantified.

Characterization and cytotoxicity assay of MSNs-rhodamine B (RhB)

The UV-Vis spectra of RhB, MSNs and MSNs-RhB were determined with a UV-Vis Spectrophotometer (Shimadzu, Japan). BMMs were seeded respectively on 96-well plates at suitable densities and allowed to adhere overnight. Different MSNs-RhB concentrations (i.e., 12.5, 25, 50, 100 μg/mL) and 30 ng/mL M-CSF were added, and the cells were cultured for 24 h. Cell viability was evaluated by Cell Counting Kit-8 (CCK-8; Dojindo, Kumamoto, Japan) and an ELISA microplate reader (Bio-Tek Instruments Inc., Winooski, VT, USA).

Lipopolysaccharide (LPS)-induced osteoclastogenesis from BMMs

BMMs were seeded on glass coverslips in 24-well plates at a density of 4×10^4 cells/well and incubated in complete culture medium with 100 ng/mL RANKL for 36 h and 1 μg/mL LPS for another 5 days. During the process, 64 μg/mL of MSNs or MSNs-ISL supplements were added. Various experiments were conducted to evaluate whether or not MSNs-ISL suppresses LPS-triggered osteoclastogenesis, including tartrate-resistant acid phosphatase (TRAP) staining, fibrous actin (F-actin) rings and lacunar resorption pits assay. The TRAP-positive cells, F-actin rings and bone resorption capacity were imaged and the statistical data were analyzed.

Supplementary table and figures

Table S1. Characterization of mesoporous silica nanoparticles (MSNs) and ISL-loaded mesoporous silica nanoparticles (MSNs-ISL).

Sample	Surface area (m^2/g)	Pore size (nm)	Zeta potential
MSNs	960.3	4.57	–30.4
MSNs-ISL	807.2	4.15	–38.2

Table S2. The specific primer sequences used for PCR.

Gene	forward	reverse
NFATc1	5′-GGTAACTCTGTCTTTCTAACCTTAAGCTC-3′	5′-GTGATGACCCCAGCATGCACCAGTCACAG-3′
c-Fos	5′-CCAAGCGGAGACAGATCAACTT-3′	5′-TCCAGTTTTTCCTTCTCTTTCAGCAGAT-3′
TRAP	5′-TGACAAGAGGTTCCAGGA-3′	5′-AGCCAGGACAGCTGAGTG-3′
MMP-9	5′-TACCCTATGTACCGCTTCAC-3′	5′-GAACAAATACAGCTGGTTCC-3′
cathepsin K	5′-CAGCAGAACGGAGGCATTGA-3′	5′-CCTTTGCCGTGGCGTTATAC-3′
GAPDH	5′-AACGGATTTGGTCGTATTGGG-3′	5′-CAGGGGTGCTAAGCAGTTGG-3′

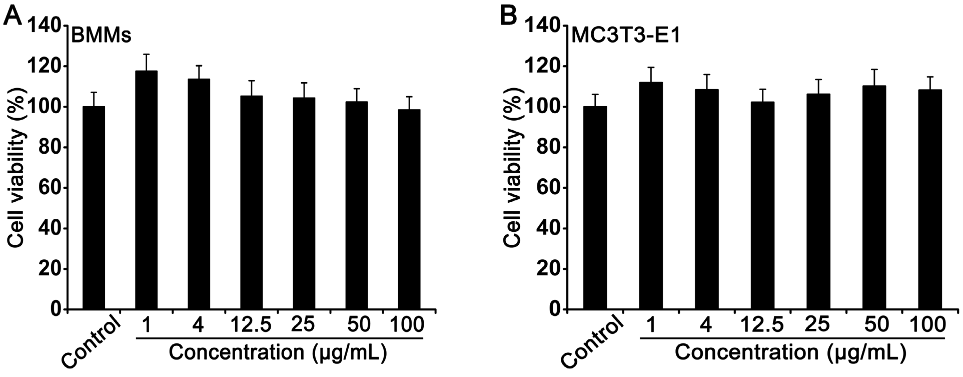

Figure S1. The cytotoxicity assay of synthesized MSNs on (A) BMMs and (B) MC3T3-E1 cells. Cells were seeded on 96-well plates at a suitable density. Different concentrations (i.e., 1, 4, 12.5, 25, 50, 100, 200 μg/mL) of MSNs were added and cultured for 24 h. Cell viability was evaluated by the CCK-8 assay.

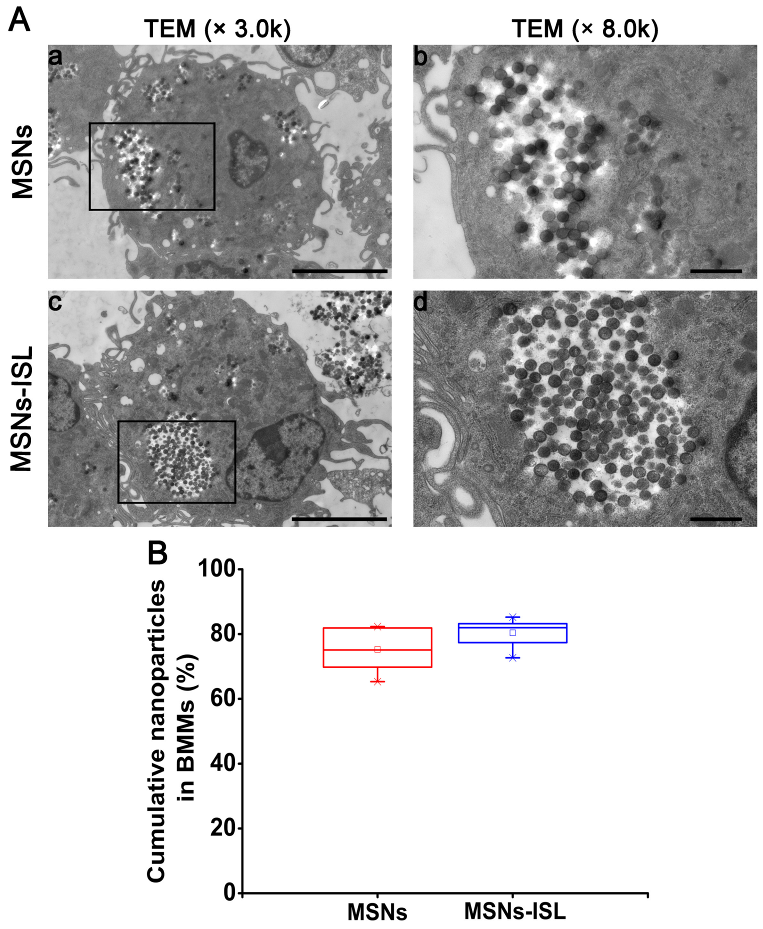

Figure S2. (A) TEM images of the endocytosis of MSNs and MSNs-ISL into BMMs after incubation for 24 h. TEM (× 3.0k): scale bar = 5 μm. TEM (× 8.0k): scale bar = 1 μm. (B) The number of nanoparticles engulfed by BMMs was calculated to explore the effects of ISL loading on cellular uptake. The endocytosis of MSNs-ISL was more than that of MSNs with an insignificant difference. $P > 0.05$ MSNs group vs. MSNs-ISL group.

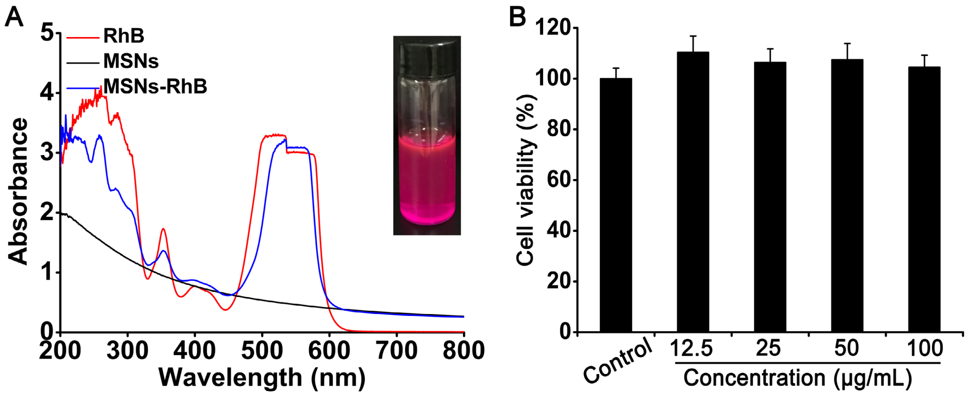

Figure S3. (A) The UV-Vis spectra of RhB (red), MSNs (black) and MSNs-RhB (blue). The image of MSNs-RhB (1 mg) in aqueous solution (2 mL). (B) The cytotoxicity assay of synthesized MSNs-RhB on BMMs. Cells were cultured with different concentrations (i.e., 12.5, 25, 50, 100, 200 μg/mL) of MSNs for 24 h. Cell viability was presented using the CCK-8 assay.

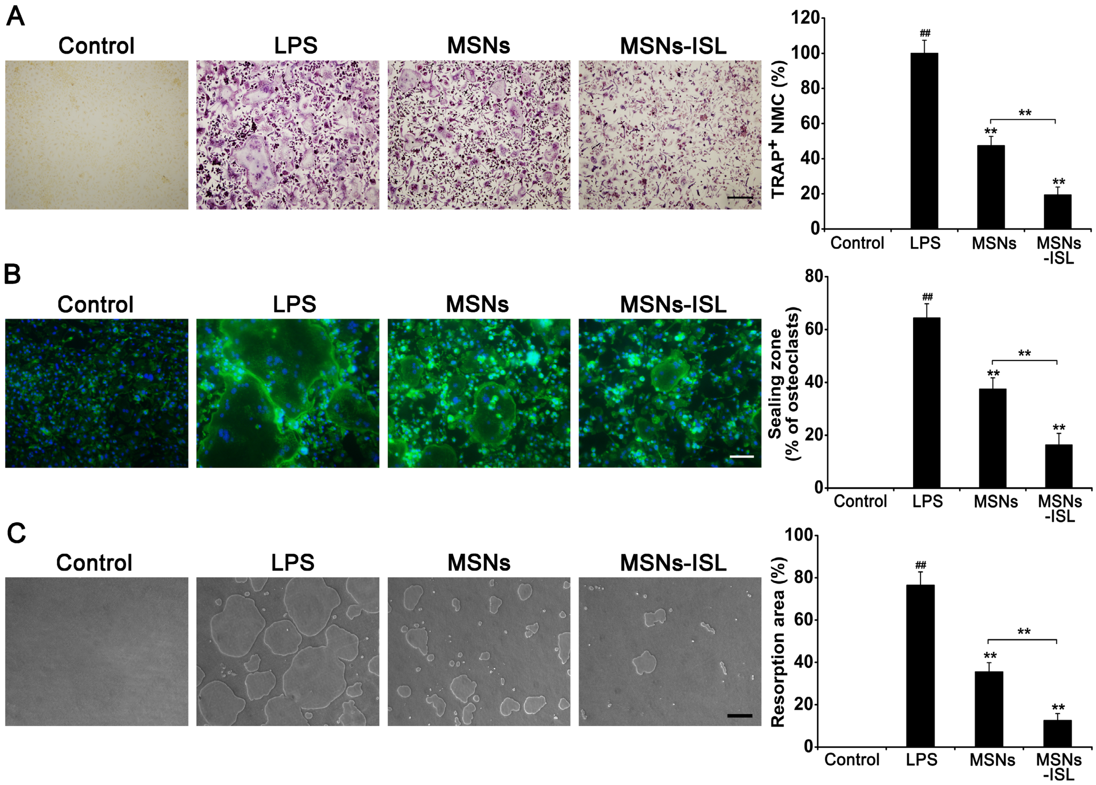

Figure S4. MSNs and MSNs-ISL suppressed LPS-stimulated osteoclastogenesis from BMMs. BMMs were cultured with 100 ng/mL RANKL for 36 h and for another 5 days with 1 μg/mL LPS. Together with the stimulator, 64 μg/mL of MSNs or MSNs-ISL were added. After cells were fixed, TRAP staining, and the F-actin rings and lacunar resorption pits assay were performed. (A) $TRAP^+$ MNCs were stained purple. Scale bar = 200 μm. $TRAP^+$ MNCs with multiple nuclei (3 or more) were counted as the percentage of positive cells in the LPS group. ## $P < 0.01$ vs. control group; ** $P < 0.01$ vs. LPS group or MSNs group vs. MSNs-ISL group. (B) F-actin rings were observed through phalloidin staining (green). Scale bar = 100 μm. The F-actin sealing zone was quantitated as percentages of total number of osteoclasts. ## $P < 0.01$ vs. control group; ** $P < 0.01$ vs. LPS group or MSNs group vs. MSNs-ISL group. (C) Bone resorption pits were observed after cells were removed. Scale bar = 200 μm. ## $P < 0.01$ vs. control group; ** $P < 0.01$ vs. LPS group or MSNs group vs. MSNs-ISL group.
